# Another dark taxon comes to light: *Semicentenialomycetes*, a new class within the *Pucciniomycotina (Basidiomycota)*, and its first described representative, *Semicentenialea rex*

**DOI:** 10.3897/imafungus.17.189848

**Published:** 2026-07-01

**Authors:** Veera Tuovinen Nogerius, Marisol Sánchez-García, Kerri Kluting, Jussi Heinonsalo, Martin Ryberg, Merje Toome, Sajeet Haridas, Stephen Mondo, Kurt LaButti, Matt Nolan, Anna Lipzen, Maxim Koriabine, Diane Bauer, Kerrie Barry, Igor V. Grigoriev, M. Catherine Aime, Anna Rosling

**Affiliations:** 1 Department of Ecology and Genetics, Uppsala University, SE-752 36 Uppsala, Sweden Department of Plant and Microbial Biology, University of California Berkeley United States of America; 2 Department of Forest Mycology and Plant Pathology, Swedish University of Agricultural Sciences, SE-750 07 Uppsala, Sweden Department of Botany and Plant Pathology, Purdue University West Lafayette United States of America; 3 Department of Forest Sciences, University of Helsinki, 00790 Helsinki, Finland U.S. Department of Energy Joint Genome Institute, Lawrence Berkeley National Laboratory Berkeley United States of America; 4 Department of Organismal Biology, Uppsala University, SE-752 36 Uppsala, Sweden Department of Forest Mycology and Plant Pathology, Swedish University of Agricultural Sciences Uppsala Sweden; 5 Department of Botany and Plant Pathology, Purdue University, West Lafayette, Indiana 47907, US Department of Forest Sciences, University of Helsinki Helsinki Finland; 6 U.S. Department of Energy Joint Genome Institute, Lawrence Berkeley National Laboratory, Berkeley, California, USA Department of Ecology and Genetics, Uppsala University Uppsala Sweden; 7 Department of Plant and Microbial Biology, University of California, Berkeley, California, USA Department of Organismal Biology, Uppsala University Uppsala Sweden

**Keywords:** eDNA, fungi, fungal culture, genomics, metabarcoding, new species

## Abstract

Only a small fraction of the global fungal diversity is described, and a large number of fungal sequences from environmental DNA (eDNA) lack relevant reference sequences for taxonomic identification in metabarcoding studies. Among these are several deeply divergent fungal lineages, hypothesized to be class- and order-level lineages, currently known only by eDNA sequences. Here, we formally describe the first representative of one such lineage, previously referred to as Clade GS25, now captured in culture. We use a phylogenomic approach to test the phylogenetic placement and taxonomic rank hypothesized for this lineage and present the formally described *Semicentenialea rex***sp. nov**. as the first known species in the novel class *Semicentenialomycetes* (*Pucciniomycotina*, *Basidiomycota*). *Semicentenialea rex***sp. nov**. was isolated from pine roots taken from a podzol soil profile in a pine forest in central Sweden and shows an affinity to mineral soil horizons in this habitat. The same species was isolated independently from pine roots collected in central Finland. Additionally, we provide the first phylogenomic resolution of *Pucciniomycotina*, using reference genomes from all described classes with the exception of *Cryptomycocolacomycetes*. In addition to expanding our knowledge about the diversity of *Pucciniomycotina*, our study highlights the importance of continued culturing efforts in combination with eDNA studies to uncover hidden fungal diversity. Furthermore, our data provide insights into the strengths and limitations associated with amplicon sequence variants, different similarity cut-offs, and genome-derived ribosomal operons.

## Introduction

Although estimates of the global fungal diversity vary, they in general exceed several million species, yet only ~150 000 species have been formally described (Niskanen et al. 2023). Consequently, the vast majority of fungal diversity remain known only from environmental DNA (eDNA) (Baldrian et al. 2022), and a large number of fungal eDNA sequences are unidentified, even to higher taxonomic ranks (Kõljalg et al. 2005; Nilsson et al. 2016). Because taxonomy is linked to knowledge about the organisms and their ecological roles, an inability to assign taxonomy to eDNA sequences is one major obstacle affecting the field of environmental mycology and ecology. It is not uncommon for a notable proportion of the operational taxonomic units (OTU) inferred in an eDNA metabarcoding study to be unidentifiable based on currently available reference sequence data, and sometimes the same unidentified DNA sequences surface repeatedly from study to study (Nilsson et al. 2016) in samples collected from across the globe (Tedersoo et al. 2014). Some of these ‘dark taxa’ known only from eDNA data (Ryberg and Nilsson 2018) represent deeply divergent lineages (Tedersoo et al. 2017). Due to their enigmatic and elusive nature, some dark taxa have even been dubbed the “50 most wanted fungi” (Nilsson et al. 2016), of which the first was identified and described in 2017 (Torres-Cruz et al. 2017). Sometimes, these unidentified eDNA sequences represent fungal lineages that remain unknown for many years despite a global distribution and often high abundance, as was the case with the class *Archaeorhizomycetes* (Rosling et al. 2011), a group previously known as “Soil Clone Group 1” (Schadt et al. 2003; Porter et al. 2008). Coincidentally, it is the investigations of *Archaeorhizomycetes* diversity that indirectly contributed to linking a fungal culture to another dark taxon. From the same field site, Kalsoom Khan et al. (2020) combined eDNA sequencing with isolation of fungi from roots into pure culture. Barcode sequencing of the ITS or the LSU region of the cultures demonstrated that none belonged to the sought-after class *Archaeorhizomycetes*. Later a search against the UNITE database revealed that among the unidentified isolates were members of Clade GS25 (corresponding to the UNITE taxon hypothesis TH069974 GS25 ord. incertae sedis) (Kõljalg et al. 2020), which was hypothesized to belong to a class sister to all other *Pucciniomycotina* (Tedersoo et al. 2017).

The *Pucciniomycotina*, known primarily for its rust fungi (*Pucciniales*), is thought to be the sister subphylum to all other *Basidiomycota* (Aime et al. 2014). Most of the subphylum, in terms of number of species, is comprised of *Pucciniales*, obligate plant pathogens (Aime et al. 2006), with characteristically complex life cycles (Aime et al. 2018a). However, in addition to *Pucciniales*, *Pucciniomycotina* contains a variety of other orders spread across ten classes. These include species with various ecological roles, ranging from endophytic (Aime et al. 2018b) to mycorrhizal with orchid host plants (Kottke et al. 2010), root-associated fungi (Bonito et al. 2017), animal associates (Aime et al. 2014), and mycoparasites (Schoutteten et al. 2024). Within *Pucciniomycotina*, many morphological traits including microscopic structures (e.g. Sugiyama et al. 2018) and dimorphic growth (Wang et al. 2015a, 2015b; Oberwinkler 2017) fail to delimit monophyletic groups. Multi-locus phylogenetic analyses have been conducted within the *Pucciniomycotina* but relationships between the major lineages are still not well resolved (Aime et al. 2006; Schell et al. 2011; Wang et al. 2015a, 2015b). Taken together, it is apparent that the *Pucciniomycotina* represents a widely diverse and species-rich group that comprises numerous unidentified organisms from lineages whose class- and order-level relationships remain unresolved. Clade GS25 is one of three novel class-level lineages identified by Tedersoo et al. (2017) within the *Pucciniomycotina* and hitherto known only from eDNA.

In this paper, we introduce a new class, *Semicentenialomycetes* class. nov., describe the first known representative of this lineage, *Semicentenialea rex* sp. nov. (*Semicentenialaceae*, fam. nov., *Semicenteniales*, ord. nov.), and present its sequenced genome, phylogenetic placement, ecology, and morphology. Further, analyses of the ribosomal operon extracted from genome data and available eDNA sequences assigned to the novel class highlight methodological challenges in using barcode sequence similarity thresholds as proxies for species.

## Methods

### Isolation, growth and sequence identification of Clade GS25 fungal cultures

Fungal cultures were isolated from surface sterilized pine root tips (healthy or dead) collected from soils sampled at Ivantjärnsheden Field Station (Jädraås, Sweden, 60°49'N, 16°30'E) in fall 2013 as part of the study by Kalsoom Khan et al. (2020). The sampled Podzol soils were separated into mineral horizons E and B. Following the method described in Menkis et al. (2014), thousands of root tips were plated on Modified Melin-Norkrans (MMN) media (Marx 1969) and incubated in the dark at room temperature. Plates were monitored regularly and roots with emerging mycelia were removed during the first two months. After this, roots with emerging mycelia were transferred to separate MMN plates for pure culturing. The 160 obtained cultures were identified by sequencing of the internal transcribed spacer (ITS) or the large subunit (LSU) using primers ITS1F (Gardes and Bruns 1993), ITS4 (White et al. 1990), and LR3 (Hopple Jr and Vilgalys 1994) MH843963–MH844060 (Kalsoom Khan et al. 2020). Based on the best ITS2 sequence matches, we discovered that our culture collection contained several isolates (Suppl. material 1: table S1) of Clade GS25 (Tedersoo et al. 2017).

All cultures with recorded sampling locations were collected from illuvial mineral soil, i.e. the B horizon of the studied Podzol soil, in two different plots. These were maintained in the Rosling lab culture collection, Uppsala University, as slant cultures and five of these, HU4064, HU4068, HU4069, HU4107, and HU4147, were grown on MMN agar plates at room temperature and examined for this study. HU4064 is also available in in the PUL culture collection, Purdue University, the Westerdjik live culture collection (CBS 150710), and Leibniz Institute DSMZ culture collection (DSM 120069). To confirm the identity of the five examined isolates, their ITS region was re-sequenced in 2018 and 2024. DNA was extracted from the cultures using the Quick-DNA™ Fecal/Soil Microbe Miniprep Kit (Zymo Research) and Plant/Fungi DNA isolation kit (Norgen Biotek Corp., Canada), respectively, following the manufacturer’s instructions. The ITS region was PCR amplified using primers ITS1F and ITS4 (White et al. 1990), and Sanger sequenced in-house on an ABI3730xl in 2018 and at Eurofins (Ebersberg, Germany) in 2024. Electrophoretogram inspection and assembly of forward and reverse reads were performed using Geneious 11.1.5 (https://www.geneious.com).

In addition, two cultures JH144 (LK052815) and JH169 (LK052826) were isolated from ectomycorrhizal roots of *Pinus sylvestris* from two different field sites in central Finland (61.8400, 24.2600 and 62.3700, 27.0700). In both cases, the Clade GS25 isolates were obtained by placing sections of surface-sterilized ectomycorrhizal pine root tips on Hagem’s agar (Modess 1941), where 35 mg/ L Streptomycin and 3 mg/L Benomyl were added. The Finnish isolates were barcoded by Sanger sequencing using the same primers as above, and those isolate cultures are available in the Microbial Domain Biological Resource Centre HAMBI Culture Collection, University of Helsinki, Finland (https://www.helsinki.fi/en/infrastructures/biodiversity-collections/infrastructures/microbial-domain-biological-resource-centre-hambi) (Suppl. material 1: table S1).

### Genome sequence data

The DNA of the isolate HU4064, labeled “*Pucciniomycotina* sp. nov. Clade gs25”, was sequenced as part of the 1000 Fungal Genomes Project (Grigoriev et al. 2014; https://mycocosm.jgi.doe.gov) at the U.S. Department of Energy Joint Genome Institute (JGI). The isolate was grown in liquid MMN in 250 ml E-flasks on a rotary table at 100 rpm at room temperature for two months. Mycelia were collected for DNA extraction using the QIAGEN Genomic-tips kit (Qiagen, Germany) following the manufacturer’s instructions. DNA extracts were sent to JGI for genome sequencing. The DNA was sheared to >10kb using Covaris g-Tubes, treated with exonuclease to remove single-stranded ends and DNA damage repair mix followed by end repair and ligation of blunt adapters using Barcoded Adapter Plate 3.0 (Pacific Biosciences). The libraries were purified with AMPure PB beads and size selected with BluePippin (Sage Science) at >10 kb cutoff size. PacBio Sequencing primer was then annealed to the SMRTbell template library, and sequencing polymerase was bound to them. The prepared SMRTbell template library was then sequenced using the Sequel II sequencer, 8M v1 SMRT cells, and Version 2.0 sequencing chemistry with 1 × 1800 sequencing movie run times.

Mitochondria-filtered subread reads were assembled with Falcon v.0.7.3 or 1.8.8 (https://github.com/PacificBiosciences/FALCON), improved with FinisherSC v.2.0 (Lam et al. 2015), and polished with Quiver version smrtanalysis_2.3.0.140936.p5. To extract and assemble the mitochondria, Falcon pre-assembled (pread) data was kmer profiled using bbtools kmercountexact.sh (https://github.com/BioInfoTools/BBMap) with default parameters, filtered with bbtools bbnorm.sh [pigz passes=1 bits=16 min=(main peak * 1.5) target=9999999], and assembled with Flye [--pacbio-corr -g 100k --asm-coverage 100] v. 2.3.6 (Lin et al. 2016). Genes were called on the initial Flye assembly using Prodigal (https://github.com/hyattpd/Prodigal) and examined for mitochondrial HMM models with hmmsearch (Eddy 1995). Contigs containing mitochondrial HMM were masked of ribosomal content with bbtools bbduk.sh [k=25 mm=f kmask=N] and used to recruit preads with bbtools bbduk.sh [k=25 mm=f mkf = 0.03 ordered ow]. Resulting preads were reassembled using Flye v.2.3.6 [-g 100k --asm-coverage 100]. A total of three rounds of read recruitment and reassembly were conducted to provide the final assembly. For the nuclear genome assembly, contigs of less than 1000 bp were excluded. JGI post-assembly analysis indicated the presence of two fungal organisms in the assembled data, and the contigs were separated into target/contaminant groups based on distinct GC content and assembly contiguity. Separation was verified and taxonomic identity determined using BLAST (Camacho et al. 2009) against NCBI RefSeq Fungi (O’Leary et al. 2016) and UNITE (Abarenkov et al. 2024), complemented by ORF-based similarity searches against NR and Mycocosm using MMSeqs2 (Steinegger and Söding 2017). Two contigs were reassigned between target and contaminant groups after their taxonomic identity was clarified by the combined BLAST and MMSeqs2 results. An additional check for contamination was done using the NCBI Foreign Contamination Screening (FCS) pipeline, which verified the absence of the contamination in the assembly of HU4064 (Astashyn et al. 2024). Mitochondrial genomes were assigned to target/contaminant assemblies based on whole-genome alignment using NUCmer (Marçais et al. 2018). The other fungal genome assembled from the reads was identified as a *Rutstroemiaceae* sp. (*Helotiales*, *Ascomycota*). We re-extracted DNA from the isolate used for genome sequencing and sequenced the ITS to verify the identity of the culture, which produced electrophoretograms that did not indicate the presence of more than one taxon in the sample. We concluded that we have a pure culture and that the second genome represents a contamination during handling of the DNA extract. This conclusion is also supported by the morphological inspection of the culture plates where no obvious contamination is visible after years of sub culturing in the laboratory, and the observation of similar cell-types and morphologies across different isolates. The genome assembly is available in the NCBI under accession JBZUJS000000000, with the raw reads in the BioProject PRJNA1466414.

The ITS sequence obtained from the isolate HU4064 via Sanger sequencing (MH843997), and the GS25 reference sequence (KY687665; Tedersoo et al. 2017) were used as BLASTn queries against the HU4064 genome assembly to locate the ribosomal operon. Raw reads were subsequently mapped to both ITS reference sequences using BWA-MEM (v. 0.7.15). In the BLASTn search, only a partial match corresponding to the ITS2 and part of the LSU region of MH843997 was detected, while the raw reads were successfully mapped to the ITS reference sequence. Therefore, we reassembled the raw reads using different genome assemblers and parameters (Suppl. material 1: table S2) and repeated the above mentioned BLASTn searches with the MH843997 sequence as a query.

### Phylogenomic analysis

To reconstruct the evolutionary relationships of Clade GS25, we assembled a dataset comprising 16 genome assemblies representing all described classes of *Pucciniomycotina*, with the exception of *Cryptomycocolacomycetes*, for which no cultured representative is known, and eight additional taxa to be used as outgroups (Suppl. material 1: table S3). These genome assemblies were either newly generated or obtained from prior publications (Padamsee et al. 2012; Cissé et al. 2013; Brown et al. 2013; Toome et al. 2014; Paul et al. 2014; Nemri et al. 2014; Albu et al. 2015; de Vries et al. 2017; Cuomo et al. 2017; Mondo et al. 2017; Mondo et al. 2025; Branco et al. 2017; Kijpornyongpan et al. 2018; Camiolo et al. 2019; Koch et al. 2021), and downloaded through the JGI genome portal (Grigoriev et al. 2014; https://mycocosm.jgi.doe.gov). For details on the DNA extractions, sequencing, genome assembly and annotation of the other *Pucciniomycotina* genomes, see Suppl. material 1: Suppl. methods.

BUSCO (v. 3.0.2b; Simão et al. 2015) with the fungi_odb9 dataset was used to extract 290 orthologs from the included genome assemblies. A dataset containing only single-copy BUSCO genes was generated; three genes were present in less than 12 taxa and were therefore excluded from subsequent analyses. Sequences corresponding to BUSCO genes were aligned using MAFFT (v.7.407; Katoh and Standley 2013) with the -auto option and trimmed using trimAl (v.1.4.1; Capella-Gutiérrez et al. 2009) with a gap threshold of 0.3, with all other parameters left at their default values. Alignments for each gene were concatenated into a supermatrix and a partition file was generated using the python script geneStitcher.py (available at https://github.com/ballesterus/Utensils). IQ-TREE (v.2.0; Nguyen et al. 2015) was used to perform model selection for each gene partition, and to determine the best partitioning scheme using ModelFinder (Kalyaanamoorthy et al. 2017). Phylogenetic inference was then conducted under a Maximum Likelihood (ML) framework (Chernomor et al. 2016), and branch support was assessed with 1 000 ultrafast bootstrap replicates. Individual gene trees were inferred using IQ-TREE, with substitution models automatically selected using the MFP option. Finally, a species tree was reconstructed using ASTRAL III (v.5.7.7; Zhang et al. 2018) which uses a coalescent-based approach.

To further evaluate and support the establishment of the proposed new class, we reconstructed a phylogenetic tree based on combined LSU and SSU rDNA sequences from representative taxa across *Pucciniomycotina* (Suppl. material 1: table S4), thereby increasing the taxon sampling and incorporating a member of the other known genus of *Classiculomycetes*, *Jaculispora submersa* (Bauer et al. 2003). Phylogenetic inference was performed using IQ-TREE with 1 000 bootstrap replicates. This analysis was conducted to assess the phylogenetic placement of HU4064 in relation to other major lineages within the subphylum and to determine whether its position was consistent with the phylogenomic results supporting the erection of a new class.

### Detection of ribosomal amplicon sequences in eDNA sequence data

To determine whether the isolated representatives of GS25 were also detected in eDNA, we searched the two amplicon datasets generated from the same study site (Kalsoom Khan et al. 2020) for ASVs that were similar to the sequences generated from the fungal isolate HU4064 (MH8439997) (Kalsoom Khan et al. 2020). For this purpose, USEARCH v.11.0.667 (Edgar 2010) was used to identify similar ASVs using ublast with the setting -acceptall. High sequence similarity was found in two long read ASVs (ASV_57: UDB0779177 and ASV_170: UDB0779801) and one short read ASV (itASV_481: MT926830). Using Geneious 11.1.4 (Biomatters Ltd) these sequences were aligned with all ITS and LSU sequences from the analyzed isolates to determine sequence similarity and detectability in ASV datasets. The ITS sequences of two cultures isolated in Finland (LK052815 and LK052826) were also included in the alignment (FigShare project https://doi.org/10.17044/scilifelab.26894242). Based on the alignment, sequence similarities between cultures and ASVs were calculated in Geneious 11.1.4 to estimate the within species sequence similarity for the ITS2 region (Suppl. material 1: table S5).

The broader distribution of the taxa was inferred based on available sequence data in the UNITE database (version 9, November 2023), using the taxon hypothesis TH069974 (GS25 ord. Incertae sedis, https://doi.org/10.15156/BIO/TH069974) as a proxy for the lineage, and the species hypothesis SH1268087.09FU as a proxy for *Semicentenialea. rex* sp. nov. To investigate the diversity of the lineage based on environmental sequences the full fungal UNITE+INSD database (v.9 2023-07-18) was downloaded (Abarenkov et al. 2024 [https://doi.org/10.15156/BIO/2938065]). All 2497 sequences annotated as GS25 were extracted based on their classification within this lineage. To reduce the dataset, all sequences were aligned using MAFFT (v.7.490; Katoh and Standley 2013) with the -linsi option, and the distance (proportion of sites that are different) between each sequence pair was calculated in Pairalign of the phylommand package (Ryberg 2016). Sequences that had zero distance sites were clustered together, and the longest sequence (disregarding ambiguous sites) was chosen as representative of the cluster using a custom Julia script. The representative sequences were realigned as before, and a maximum likelihood phylogenetic tree was estimated in IQ-TREE (v.2.0.7; Nguyen et al. 2015) using the best fitting model according to the BIC criteria and using the fast tree search algorithm. In the absence of a suitable outgroup, the phylogeny was mid-point rooted. The maximum distance within each UNITE species hypothesis (USH) and minimum distance between each pair of USH was identified using a custom Julia script. The Julia scripts are available in the FigShare project (https://doi.org/10.17044/scilifelab.26894242.)

### Morphological studies with conventional and confocal laser scanning microscopy

Isolates HU4064, HU4107, HU4147, JH144, JH169, grown on the growth media described above, were studied for their general morphology with conventional light microscopy with Leica DM2500 LED microscope, HC PL APO 63x/1.50–0.60 oil objective and DIC, fitted with Leica K3C color camera and annotated with LasX software (Leica) in black-and-white mode. At the time of microscopical observation, the Swedish cultures were approximately four months old and the Finnish cultures almost two years old.

In addition to the conventional light microscopy, isolates HU4064, HU4068, and HU4147 were studied with confocal laser scanning microscopy (CLSM). The isolates were grown for three to eight weeks (in one case 8.5 months) on MMN agar at 21 °C. Small mycelial samples were collected from the plates and fixed in 70% ethanol for 10 minutes and washed with phosphate-buffered saline (PBS) buffer, pH 7.4. Next, the samples were stained with 1 µg/mL propidium iodide (PI; ThermoFisher Scientific) in order to stain the nuclei and then washed with PBS. Samples were then stained with 1 g/L Calcofluor White (Honeywell Fluka, ThermoFisher Scientific) or 5 μM Calcofluor White (Biotium) and washed twice with PBS buffer. Stained samples were mounted in SlowFade™ Diamond Antifade Mountant (Invitrogen™, ThermoFisher Scientific). Microscopy was conducted on a Zeiss LSM700 or LSM710 confocal microscope with a plan apochromat 63× NA 1.4 oil M27 objective at Biological Visualisation platform (BioVis, Uppsala University, Uppsala, Sweden) or with Leica Stellaris 5 with a plan apochromatic 40× NA 1.25 glycerol objective at the Competence Center for Hidden Biodiversity, Uppsala University. Laser lines 405 nm and 488 nm were used for excitation of Calcofluor White and PI, respectively, and the respective detection wavelengths were 410–523 nm and 566–797 nm with the Zeiss microscope. With Leica Stellaris, laser lines 405 nm and 549 nm were used for excitation of calcofluor white and propidium iodine, respectively, and their emission was detected on 430–539 nm and 594–725 nm. Sequential scanning was used when acquiring z stacks. Maximum intensity projections of the image stacks were produced using Fiji (Schindelin et al. 2012, 2015) and 3D rendering with Zen 2.6 Blue (Zeiss). Color brightness and contrast were adjusted for clarity of presentation.

In addition, mycelial growth rate at room temperature was measured along four radii of the expanding colonies by marking the mycelial front at three time points over 1.5 months of growth for the isolates HU4064, HU4069, HU4069, HU4107, and HU4147.

## Results

### Genome assembly and phylogenomic analyses

The JGI assembly of the HU4064, after removing reads from *Rutstroemiaceae* sp., consisted of 54,259,369 bp across 405 scaffolds with a CEGMA capture (Parra et al. 2007) of 99,13% (18,50% unique) and a BUSCO (Simão et al. 2015) completeness of 92%.

**Figure 1.**
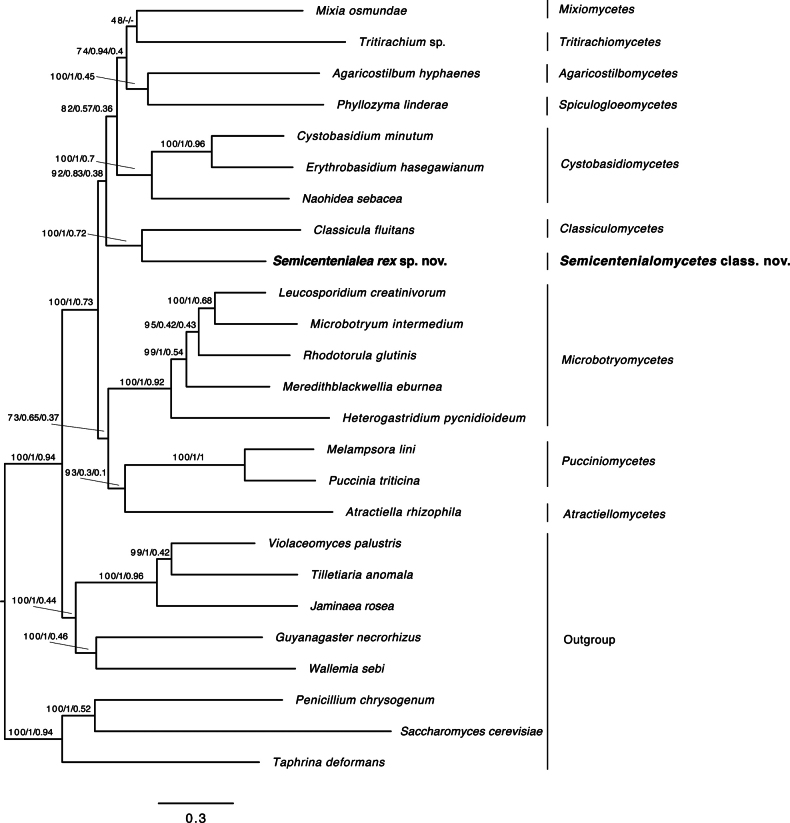
Phylogenetic placement of HU4064. Best maximum likelihood (ML) tree from a concatenated alignment of 287 BUSCO genes shared among at least 12 taxa. The numbers on the branches indicate bootstrap support (BS) from the ML analysis, local posterior probabilities (LPP) and quartet scores from the ASTRAL analysis (BS/LPP/quartet score). Missing values indicate no support for that relationship in the ASTRAL analysis. The tree includes 16 taxa of *Pucciniomycotina*, *Semicentenialea rex* sp. nov. (HU4064) previously known as GS25, as well as eight outgroup taxa representing the subphyla *Ustilaginomycotina* (*Basidiomycota*; *Tilletiaria anomola*, *Violaceomyces palustris*, *Jaminaea rosea*), *Agaricomycotina* (*Basidiomycota*; *Wallemia sebi*, *Guyanagaster necrorhizus*), *Saccharomycotina* (*Ascomycota*; *Saccharomyces cerevisiae*), *Taphrinomycotina* (*Ascomycota*; *Taphrina deformans*), and *Pezizomycotina* (*Ascomycota*; *Penicillium chrysogenum*).

Based on 287 conserved genes recovered across the included taxa, we resolved Clade GS25 within the *Pucciniomycotina* (bootstrap support (BS) from the ML analysis = 100%, local posterior probabilities (LPP) from the ASTRAL analysis = 1.0), as sister to *Classiculomycetes* (represented by *Classicula fluitans*) (BS = 100%, LPP = 1.0) (Fig. 1), and propose *Semicentenialomycetes* class. nov. for this lineage. In our analysis, all lineages for which more than one representative was sampled are well-supported: *Cystobasidiomycetes* (BS = 100%, LPP = 1.0), *Microbotryomycetes* (BS = 100%, LPP = 1.0), and *Pucciniomycetes* (BS = 100%, LPP = 1.0) (Fig. 1), as well as the node separating *Spiculogloeomycetes* and *Agaricostilbomycetes* (BS = 100%, LPP = 1.0). The topologies from the ML and ASTRAL analyses were not identical, but their differences did not affect the placement of *Semicentenialomycetes* class. nov. (Fig. 1, Suppl. material 1: fig. S1). The two topologies differ in the placement of *Atractiellomycetes* (represented by *Atractiella rhizophila*) which is recovered as sister to *Pucciniomycetes* in the ML analysis with a bootstrap support (BS) of 93% (Fig. 1) and as sister to *Microbotryomycetes* in the ASTRAL tree with a local posterior probability (LPP) of 0.65 (Suppl. material 1: fig. S1). The second difference is the placement of *Tritirachiomycetes* (represented by *Tritirachium* sp.). In the ML analysis, it is recovered as sister to *Mixiomycetes* with a BS of 48% (Fig. 1) and in the ASTRAL analysis it is inferred as sister to *Cystobasidiomycetes*, *Agaricostilbomycetes*, *Spiculogloeomycetes*, and *Mixiomycetes* with an LPP of 1.0 (Suppl. material 1: fig. S1).

Relationships between most *Pucciniomycotina* classes have been resolved in this analysis, but many without strong support (i.e., BS ≥ 99 and LPP ≥ 0.8). The class-level lineages within the *Pucciniomycotina* split into two clades: one containing *Mixiomycetes*, *Tritirachiomycetes*, *Agaricostilbomycetes*, *Spiculogloeomycetes*, *Cystobasidiomycetes*, *Classiculomycetes*, and *Semicentenialomycetes* (BS = 92%, LPP = 0.83), and a sister clade containing *Microbotryomycetes*, *Pucciniomycetes*, and *Atractiellomycetes* (BS = 73%, LPP = 0.65) (Fig. 1). Within the first clade, a clade containing *Mixiomycetes*, *Tritirachiomycetes*, and *Agaricostilbomycetes* is recovered (BS = 74%, LPP = 0.94), as sister to *Cystobasidiomycetes* (BS = 82%, LPP = 0.57) (Fig. 1). There is also support for a clade containing *Classiculomycetes* and *Semicentenialomycetes* (BS = 100%, LPP = 1.0) that is sister to the clade containing *Mixiomycetes*, *Tritirachiomycetes*, *Agaricostilbomycetes*, and *Cystobasidiomycetes* (BS = 92%, LPP = 0.83). Within the second clade, the *Atractiellomycetes* is recovered as sister to the *Pucciniomycetes* (BS = 93%, LPP = 0.3), which in turn is recovered as sister to the *Microbotryomycetes* (BS = 73%, LPP = 0.65) (Fig. 1).

The phylogenetic tree based on LSU and SSU sequences from multiple representatives of *Pucciniomycotina* (Suppl. material 1: Suppl. material. S2) shows that *Classicula fluitans* and *Jaculispora submersa* (members of the two known genera in *Classiculomycetes*) form a well-defined clade which is distinct from *Semicentenialea rex* sp. nov. The LSU–SSU tree does not provide strong support for the placement of *S. rex* sp. nov, but its position is more robustly resolved in the phylogenomic analysis (Fig. 1). Together, these results provide support for the establishment of a new class.

### Ribosomal operon sequences from cultures and environmental samples

In the JGI genome assembly, we recovered a partial ITS sequence that showed 98% sequence similarity to that of the Sanger sequences from the ITS2 extending into the beginning of the LSU region. However, the SSU, ITS1, and 5.8S regions were not recovered in the assembly. Examination of raw read mappings across two Sanger sequences, one from the same isolate (MH843997) and the GS25 reference sequence (KY687665) from (Tedersoo et al. 2017), indicated that the full-length ITS sequence was well supported by the reads, even though the assembly failed to reconstruct a complete and accurate representation of the region. Interestingly, using certain genome assemblers, we were able to reconstruct the full ITS region with 99% similarity to MH843997, although the recovered copy number and sequence similarity varied depending on the assembler and parameter settings (Suppl. material 1: table S2).

The exact rDNA sequence recovered by PCR and Sanger sequencing from the isolate HU4064 was not captured by the ASVs generated from eDNA samples concomitantly collected from the field site. Due to occasional transitions and a homopolymer sequencing error, similarity across the ITS2 region between the different ASVs and the Sanger sequenced ranged between 97.4–98.7 (Suppl. material 1: table S5). This corresponds to 4–8 bp over the 300 bp length of the ITS2. These differences could represent biological differences but are likely the result of amplification and the use of different sequencing technologies (including IonTorrent, PacBio and Sanger in this case). Across the isolates in our collection, two (HU4120 and HU4150) generated LSU sequences identical to one of the eDNA long reads (UDB0779801: ASV_170) which also had an ITS2 sequence identical to the short read eDNA ASV (MT926830: itASV_481). No isolates presented rDNA sequences identical to the second eDNA long read ASV from the study site. Although none of the rDNA sequences from the cultures were identical for the entire ITS2 region or the sequenced part of the LSU, they were all highly similar, and we are confident that they represent the same species. The two cultures from Finland had ITS regions with above 99% sequence similarity to the Swedish cultures (Suppl. material 1: table S5).

In the version 9 of the UNITE database the GS25 lineage is represented by the taxon hypothesis TH069974, which encompass 2497 sequences annotated as GS25 in UNITE+NCBI. These clustered into 19 USH at the 1.5% dissimilarity threshold. Ten are singleton USHs, and only three of those with more than one sequence are detected in more than one study (Suppl. material 1: table S6). Thus 16 of the USHs represent single observations which may or may not be biologically meaningful, suggesting that the three remaining USHs are most likely to represent different species in the lineage. One of these stands out as the most frequently observed, SH1268087.09FU, which encompasses 2681 ITS sequences captured in samples from all continents except Africa and Antarctica (https://doi.org/10.15156/BIO/SH1268087.09FU). This USH includes ITS sequences from both Swedish and Finnish isolates of *S. rex* sp. nov., as well as ASVs generated by Kalsoom Khan et al. (2020) and analyzed in this study. In SH1268087.09FU, the majority of the sequences were amplified from soil eDNA samples, with few exceptions including amplification from leaves of *Larix olgensis* (UDB0749737) and *Quercus ilex* (UDB0753573), and root tips of *Lithocarpus densiflorus* (DQ273362). We use the metadata associated with sequences captured by SH1268087.09FU to estimate the habitat and geographic distribution of *S. rex* sp. nov. (Suppl. material 1: fig. S7).

After clustering GS25 annotated sequences on zero distance, 1426 unique sequences were aligned. Visual inspection of the GS25 sequence alignment clearly showed that there were three main groups of sequences that did not align well to each other, and formed separate clades in the tree (Suppl. material 1: Figs S8, S9). One of these major clades was dominated by sequences from SH1268087.09FU, with 2443 GS25 annotated sequences (1392 after clustering zero distance sequences) including the sequences from the *S. rex* sp. nov. isolates examined in this study and multiple eDNA sequences identical to the different isolates. Ten other USHs clustered together with SH1268087.09FU in the tree, forming clade 1 (Suppl. material 1: table S6). The sequences within the clade 1 had a maximum pairwise distance of 0.055 (≥94.5% similarity). At higher dissimilarity thresholds, the USHs in clade 1 all group together into one USH, SH0499738.09FU at the 2.5% dissimilarity and into SH0215652.09FU at 3%. We conclude that the 11 USHs in clade 1 represent *S. rex* sp. nov.

The remining eight GS25 USHs formed two distinct clades in the tree (Suppl. material 1: fig. S8; table S6). While the species diversity within the lineage remains to be explored, it is likely that there are at least three species in the class. Sequences representing *S. rex* sp. nov. can be distinguished from sequences of the other two, yet unidentified, taxa in the class by matching to a 21 bp diagnostic sequence corresponding to bases 181–201 in MH843997 (Suppl. material 1: fig. S9). All cultures and ASVs analyzed in this study are identical across the region and by allowing for 1 bp mismatch all unique sequences in clade 1 match the region and can be distinguished from all unique sequences in clades 2 and 3.

Reads representing *S. rex* occur at low abundance in the concomitantly analyzed soil samples as indicated by reads clustered into itASV_481 (MT926830) in the eDNA dataset that was used for ecological inference (Kalsoom Khan et al. 2020). This ASV was detected at low relative read abundance (4.8% in one sample, but 0.3% or less in the rest) in 22 of the 24 mineral soil samples (E and B soil horizons), and in extremely low sequence read abundance in two of the 12 organic soil samples (0,005% and 0,009%) suggesting a preference for mineral soil horizons at the site.

### Taxonomy

#### Semicentenialomycetes

Taxon classificationAnimaliaSemicentenialesSemicentenialaceae

Rosling, Aime & Kluting class. nov.

B14E944E-8C57-5C85-BE18-3153E58B9187

Index Fungorum: IF904735

##### Diagnosis.

Members of *Pucciniomycotina*, similar to *Classiculomycetes* but lacking tremelloid haustorial cells and Ingoldian conidia, and found in soils.

##### Type.

*Semicenteniales* Rosling, Aime & Kluting, ord. nov., this paper

##### Description.

Dikaryotic hyphal fungi, lacking conspicuous fruiting bodies or pigmentation.

##### Distribution and ecology.

Found in soils worldwide.

##### Note.

This class was erected to accommodate the species *Semicentenialea rex* sp. nov. and encompass the lineage of unidentified fungi referred to as ‘Clade GS25’ (Tedersoo et al. 2017). *Classiculomycetes* is the closest known related lineage to *Semicentenialomycetes*, and the two are morphologically and ecologically distinct. Species within *Classiculomycetes* do not exhibit a yeast form, they have tremelloid haustorial cells and possess conidia that have two, three, or four long subapical appendages (Marvanová and Bandoni 1987; Bauer et al. 2003; Aime et al. 2014; Qiao et al. 2018). Of the three known species, only in *C. fluitans* a sexual stage has been observed on the water surface and basidia have characteristically subapically swollen sterigmata (Aime et al. 2014). Both filamentous and yeast forms could be observed in *Semicentenialea rex* sp. nov. and neither tremelloid haustorial cells nor multi-radiate conidia were observed. Further, basidium-like cells that lacked subapically swollen sterigmata could be observed. Ecologically, the *Classiculomycetes* is associated with aquatic habitats (Bauer et al. 2003; Aime et al. 2006), particularly leaf litter in freshwater habitats (Aime et al. 2014; Qiao et al. 2018), whereas *Semicentenialomycetes* has been isolated from root tips and detected as DNA sequences in soil samples from around the globe (Tedersoo et al. 2014, 2017).

#### Semicenteniales

Taxon classificationAnimaliaSemicentenialesSemicentenialaceae

Rosling, Aime & Kluting ord. nov.

7CBD607D-5D51-573C-9C35-84DEE858FC3E

Index Fungorum: IF904734

##### Diagnosis.

Similar to *Classiculales* but terrestrial and lacking tremelloid haustorial cells and Ingoldian conidia.

##### Type.

*Semicentenialaceae* Rosling, Aime & Kluting, fam. nov., this paper.

##### Description.

Same as that for *Semicentenialomycetes*.

#### Semicentenialaceae

Taxon classificationAnimaliaSemicentenialesSemicentenialaceae

Rosling, Aime & Kluting fam. nov.

B3DE28E1-0542-5E4F-BEC7-909AF24D5D1C

Index Fungorum: IF904736

##### Diagnosis.

Similar to *Classiculaceae*, but terrestrial and lacking tremelloid haustorial cells and Ingoldian conidia.

##### Type.

*Semicentenialea* Rosling, Aime & Kluting, gen. nov., this paper.

##### Description.

Same as that for *Semicenteniales*.

#### Semicentenialea

Taxon classificationAnimaliaSemicentenialesSemicentenialaceae

Rosling, Aime & Kluting gen. nov.

358F35BF-E574-5BE3-976C-3C789DA886C6

Index Fungorum: IF901103

##### Diagnosis.

Dimorphic, hyaline, dikaryotic fungi lacking fruiting bodies, tremelloid haustorial cells and Ingoldian conidia.

##### Type.

*Semicentenialea rex* sp. nov. Rosling, Aime & Kluting, this paper.

##### Etymology.

Latin for semicentennial, or fifty-year anniversary, in honor of the golden jubilee of Carl XVI Gustaf, King of Sweden. Throughout his reign, King Carl XVI Gustaf has worked tirelessly for biodiversity conservation and sustainable use of natural resources, especially within boreal forest ecosystems.

##### Description.

In culture, hyphae hyaline, septate, clamped, giving rise to obovoid thin-walled basidium-like cells and probasidium-like cells. Also producing intercalary chlamydospore-like swellings, demarcated by septa.

##### Habitat and distribution.

Associated with roots of *Pinus sylvestris* in illuvial mineral soil layers of a Podzol profile. The genus (as estimated by UNITE TH069974) has a broad geographic distribution and has been detected as environmental DNA sequences primarily in soils from North America, South America, Australia, Asia and Europe.

##### Note.

*Semicentenialea* is erected to accommodate the new species *Semicentenialea rex* sp. nov. as the first representative of the lineage of unidentified fungi previously referred to as ‘Clade GS25’ (Tedersoo et al. 2017) and currently recognized in the UNITE database as TH069974 (https://doi.org/10.15156/BIO/TH069974). The eDNA records in UNITE vs.9 indicate that there are likely at least three species in the genus, although thus far only *S. rex* sp. nov. has been recovered in culture.

#### Semicentenialea rex

Taxon classificationAnimaliaSemicentenialesSemicentenialaceae

Rosling, Aime & Kluting sp. nov.

40124450-514C-5453-92F5-E007805DB55A

Index Fungorum: IF901104

Figs 2, 3, 4

##### Diagnosis.

All cultured strains are separated from other taxa, currently known only from eDNA, in the class by ribosomal sequences possessing the following characters in ITS2: CCTGTTTGAGTGTCATAATAC at position corresponding to bases 181–201 in MH843997.

##### Holotype.

SWEDEN • Jädraås, Ivantjärnsheden Field Station (60.8145, 16.5070, altitude 185 m), from surface-sterilized root tips of *Pinus sylvestris* (*Pinaceae*), August 2013, H. Urbina HU4064 (Holotype: UPS collection F-1085350, as dry metabolically inactive culture; ITS = GenBank MH843997.

##### Ex-type cultures in culture collections.

CBS 150710 (Netherlands), HU4064 (PUL, USA), DSM 120069 (DSMZ, Germany), HU4064 (Rosling lab, Sweden).

##### Etymology.

Latin, *rex*, for king, referring to King Carl XVI Gustaf, King of Sweden.

##### Description.

**Figure 2.**
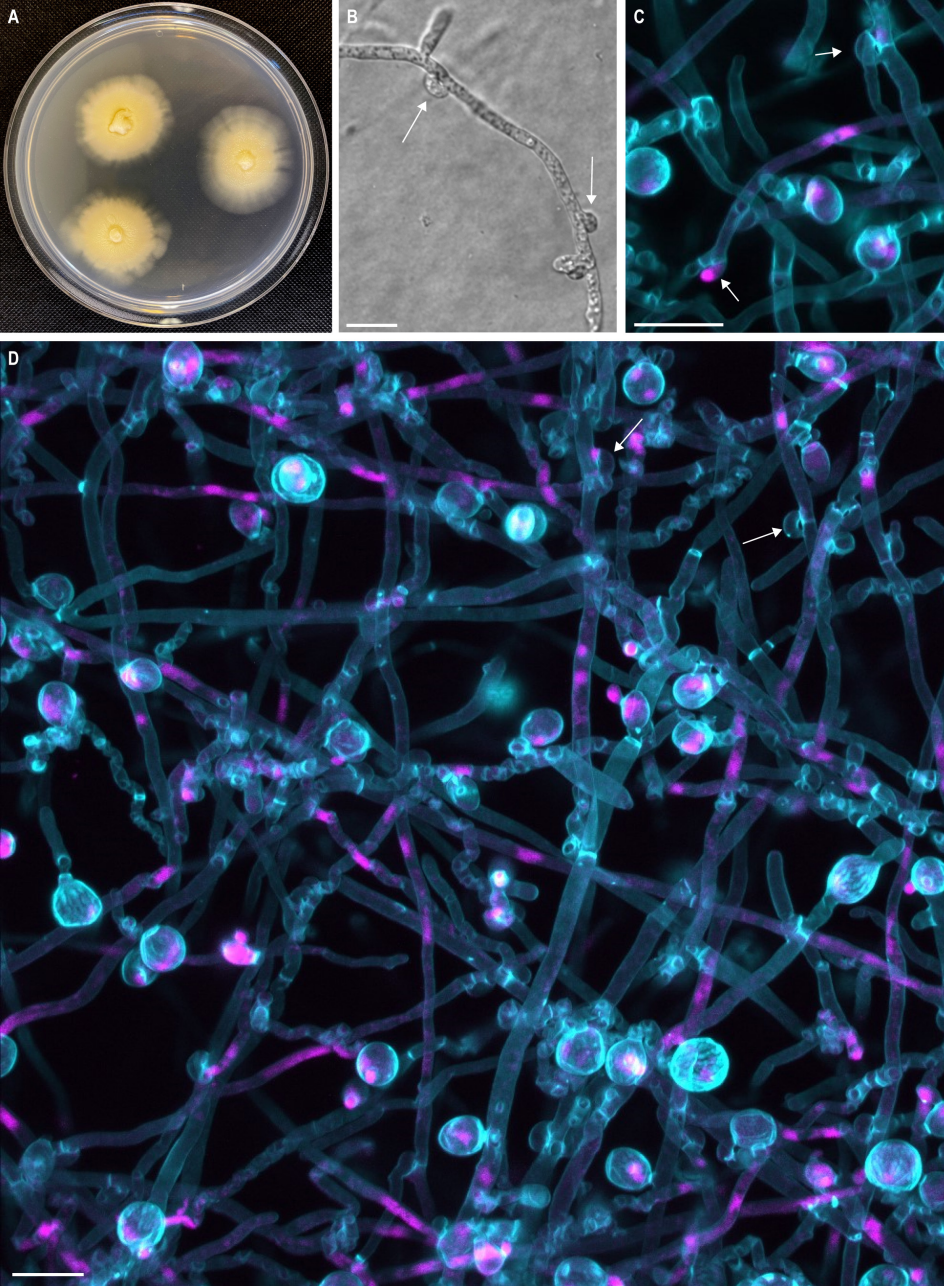
Macro- and microscopic characters of *Semicentenialea rex* sp. nov. Nuclei stained with propidium iodine (magenta) and cell walls and septa with calcofluor white (cyan), maximum intensity projection of a z-stack in C–D. **A** Colony growth after eight weeks (petri dish diameter = 90 mm; HU4064, type). **B** Hypha with clamp connections (arrows) (JH144). **C** Hyphae with clamp connections (arrows), one with a nucleus in the forming clamp (HU4064, type). **D** Overview of the hyphae with clamp connections (arrows) and nuclei. Different developmental stages of three types of swellings: basidium-like cells (hereinafter BLC), probasidium/teliospore-like cells (hereinafter TLC), and intercalary swelling can also be seen (HU4064, type), for detailed description of these structures see Figs 3, 4. Rotating 3D rendering of D as Suppl. material 2: movie S1. Scale bars 10 μm.

Colonies extending at 0.3–0.4 mm per day at room temperature on modified MMN media. Colonies pale ochre towards the center, becoming lighter and more diffuse near the margin with no reverse color when grown for eight weeks at ambient room temperature (Fig. 2A). Towards the center, colonies appearing opaque, damp, slightly glistening, leathery to slightly wrinkly, and slightly raised from the agar; producing aerial mycelium occasionally. Margin somewhat irregular, appearing more filamentous, more effuse, translucent, and slightly submerged. Hyphae hyaline, septate, producing clamp connections (Fig. 2B–D, Suppl. materials 2, 3: movies S1, S2); primarily binucleate, but occasionally multi-nucleate (Fig. 2C, D, Suppl. material 1: fig. S3, Suppl. materials 2, 3: movies S1, S2), 1–1.5 mm in diameter; producing no detectable odors.

**Figure 3.**
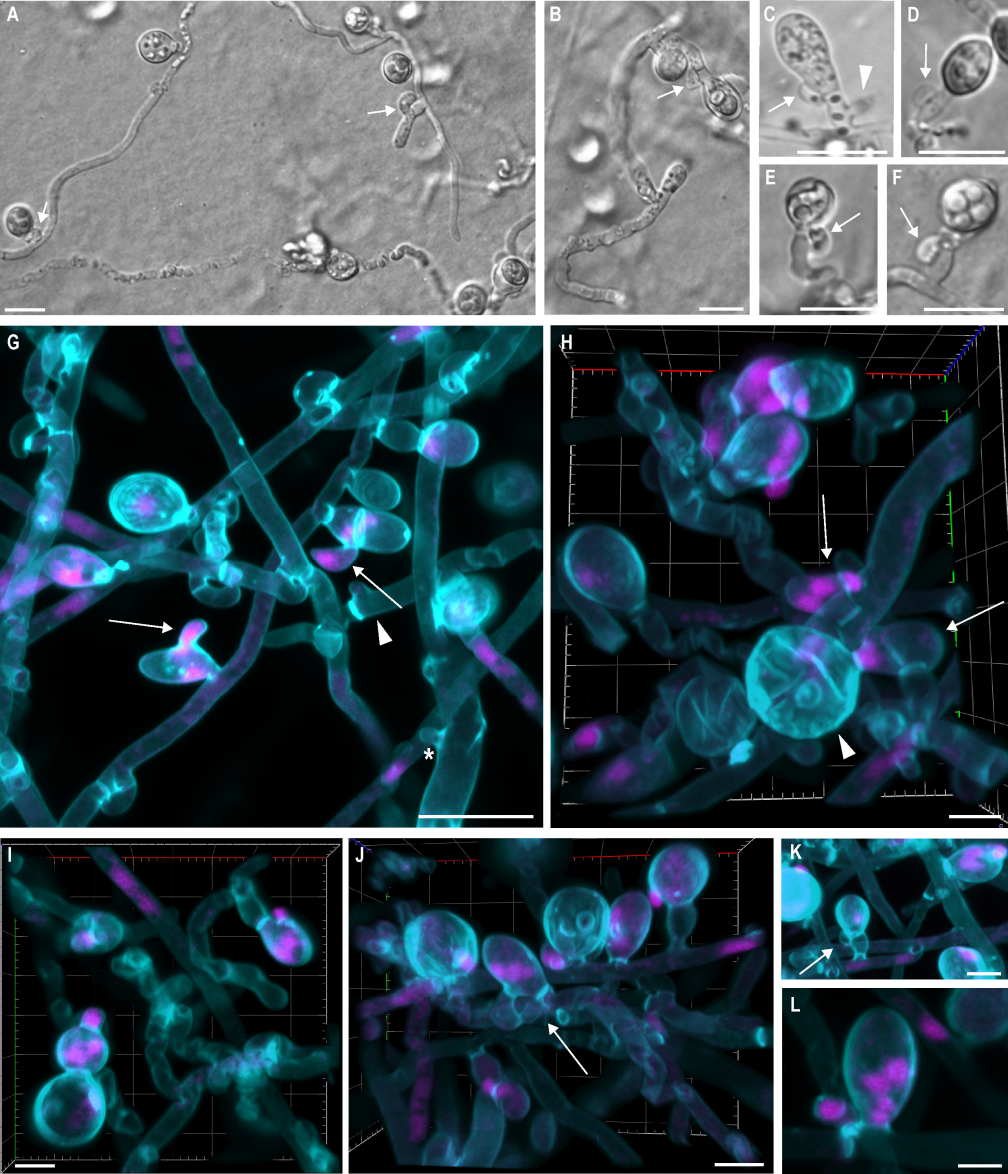
Variation in the morphology of the basidium-like cells (BLC) in *Semicentenialea rex* sp. nov. **G–L** 3D volume renderings of z-stacks, nuclei stained with propidium iodine (magenta) and cell walls and septa with calcofluor white (cyan). **A–F** BLCs with basal clamp connections (arrows). **C** A lateral cell arising from the BLC stalk (arrowhead). **G** BLCs with lateral cells with one nucleus (arrows), one BLC with a ring of strong calcofluor white staining in the obovoid cell and one nucleus in each department. Clamp connection forming (arrowhead), and yeast cells budding from hyphae indicated with strong calcofluor white staining at the base (asterisk). **H**BLC formed from a thick-walled probasidial-like swelling (TLC) (arrowhead), forming a second BLC with two nuclei in the obovoid cell and one in the lateral cell (arrow). Rotating 3D rendering of H as Suppl. material 4: movie S3. **I** Two globose BLCs in a string. **J** Lateral cell of a BLC anastomosing to the generative hypha (arrow), three nuclei in the obovoid cell. **K**BLC with a basal clamp connection (arrow). **L** Possible early stage of a BLC with four nuclei. Isolate HU4107 (**A**, **C–F**); JH144 (**B**), HU4064, type (**G–L**). Scale bars 10 μm (**A–G**); 4 μm (**H–I**, **K**); 5 μm (**J**); 3 μm (**L**).

Hyphae producing three types of swellings: basidium-like cells (hereinafter BLC), intercalary inflated cells, and probasidium/teliospore-like cells (hereinafter TLC) (Fig. 2D, Suppl. material 1: fig. S5), all often occurring simultaneously on a small area of the culture. BLCs obovoid, thin-walled, emerging directly or on a short stalk from generative hyphae, subtended by a clamp connection (Fig. 3A–L, Suppl. material 1: fig. S4A–D, F–H), with two nuclei in the obovoid cell, giving rise to a lateral cell with one nucleus (Fig. 3G–K). The obovoid BLC sometimes with a ring with a strong calcofluor white staining, indicating a possible separation of the cell to two in later developmental phases (Fig. 3G–H). Four nuclei occurring in a putatively initial phase of the BLC formation(Fig. 3L). The obovoid BLCs occasionally arising from TLCs (Fig. 3H, Suppl. material 4: movie S3).

**Figure 4.**
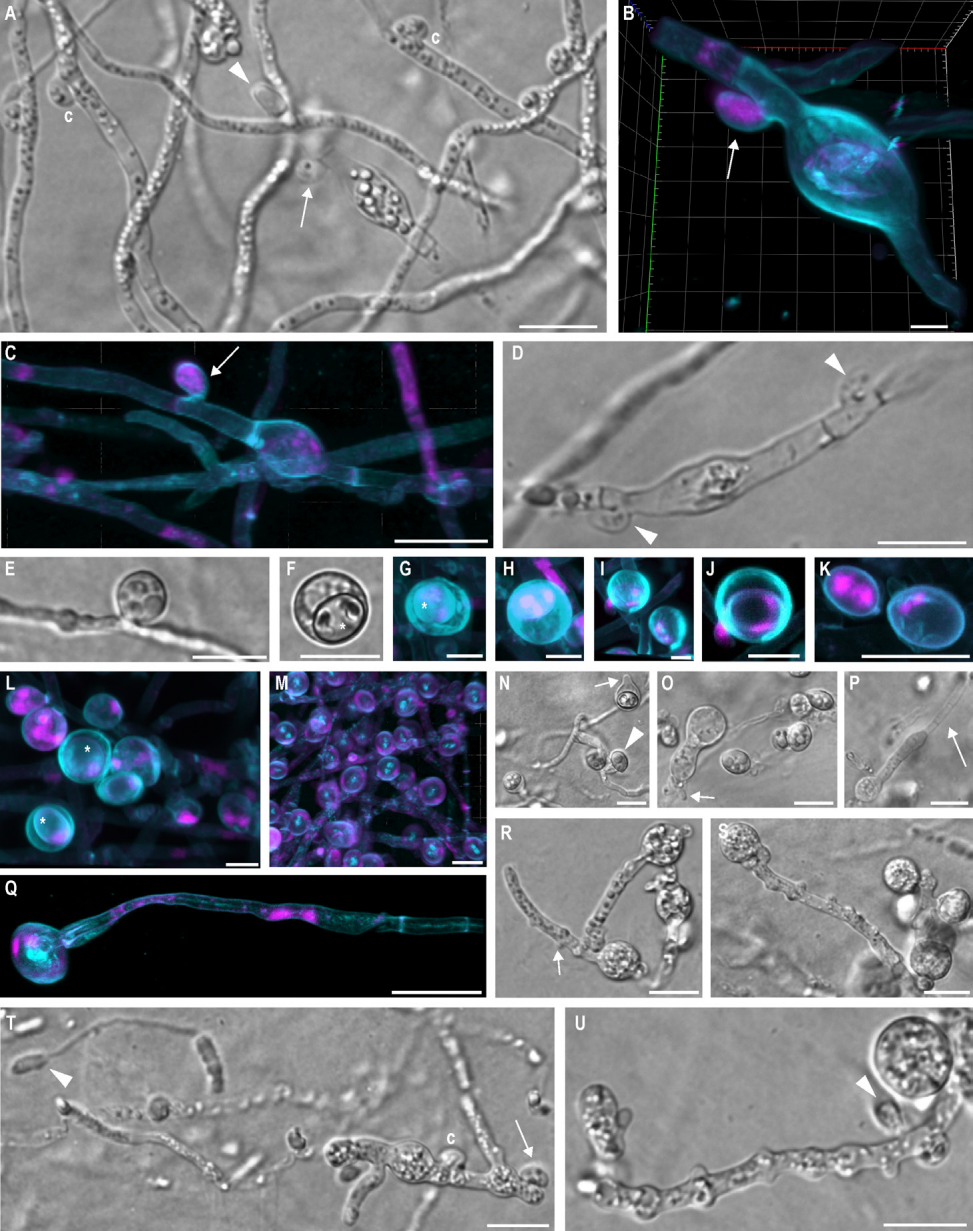
Intercalary and probasidium/teliospore-like (TLC) swellings and promycelium-like hyphae in *Semicentenialea rex* sp. nov. Nuclei stained with propidium iodine (magenta) and cell walls and septa with calcofluor white (cyan). 3D volume rendering of z-stacks in **B–C**, **G–I**, **K–M**. Maximum intensity projection in **Q**. **A** An intercalary swelling separated by two septa, with a potential cell budding of close to one of them (arrow). Short terminal cell at the end of the same hypha (arrowhead). **B** A budding cell close to an intercalary swelling with one nucleus (arrow). **C** An intercalary swelling separated by septa on both sides. A budding cell (arrow) from the adjacent cell to the intercalary swelling. **D** Intercalary swelling with clamps forming on both sides (arrowheads). **E** A thick-walled, sessile probasidium/teliospore-like swelling. **F–G** Detached TLC with a spore-like cell forming inside (asterisk), with two nuclei in **G**. **H** TLC with a spore-like cell with three nuclei. **I** TLC attached to the generative hypha with budding cell with one nucleus, and two nuclei inside the spore-like cell. **J** One optical section of I showing the binucleate spore-like cell inside the TLC. **K** Binucleate spore-like cells released from TLCs. **L** TLCs in different developmental stages, with the spore-like cells more visible in some cells (asterisk). **M** Possibly early developmental stages of TLCs with two spots staining strongly with calcofluor white, hypothetical initial stages of the cell-wall of the spore-like cell. Rotating 3D rendering of M as Suppl. material 6: movie S5. **N** Detached TLC starting to germinate (arrow) and a released spore (arrowhead) **O** TLC germinating and forming a thick promycelium-like hypha, with possible conidial cells at one end (arrow). **P** Promycelium and thin, septate hypha (arrow). **Q** Terminal TLC and dikaryotic hyphae. **R–S** Knobbly promycelium with possible small yeasts budding of (arrow). **T** Promycelium with clamp connection (c) and budding cell (arrow). A single cell germinating (arrowhead). **U** Yeast cell (arrowhead) budding of from a knobbly promycelium. Isolate HU4064, type (**A–D**, **G–L**, **T**); HU4107 (**E–F**, **N–P**); HU4147 (**M**, **Q**); JH144 (**R–S**, **U**). Scale bars: 10 μm (**A**, **C–F**, **K**, **M–P**, **Q–U**); 5 μm (**B**, **G**, **J**, **L**), 4 μm (**H**), 3 μm (**I**).

Intercalary inflated cells typically separated by a septum on either side, sometimes with budding structures near the septa (Fig. 4A–D, Suppl. material 1: fig. S5). TLCs globose, thick-walled (Fig. 4E–S, Suppl. material 1: figs S5, S6, Suppl. material 5: movie S4), especially frequent in the older parts of the culture, but also present intermixed with the BLC cells close to the growth edge. A spore-like cell developing inside TLCs with two or three nuclei (Fig. 4F–K), being released when the TLC cell walls degrade (Fig. 4K, N). Inside the TLCs, spot-like structures stain strongly with calcofluor white, which we interpret as being related to the early stage of the cell wall development of the spore-like cells (Fig. 4M). TLCs frequently giving rise to a lateral cell with one nucleus (Fig. 4I, J, Suppl. material 1: fig. S3B). TLCs detaching from the hyphae and germinating (Fig. 4N–U), producing knobby promycelial-like hyphae (Fig. 4U).

##### Habitat and distribution.

Associated with roots of *Pinus sylvestris*, but environmental sequences captured by SH1268087.09FU indicate that the species also occurs in soils where other potential host plants dominate. Near global distribution based on eDNA sequences captured by SH1268087.09FU with records from North America, South America, Australia, Asia and Europe.

##### Additional material examined.

SWEDEN • Jädraås, Ivantjärnsheden Field Station (60.8145, 16.5070, altitude 185 m), surface sterilized root tips of *Pinus sylvestris* (*Pinaceae*), August 2013, H. Urbina HU4068 (PUL culture collection; ITS = GenBank PQ270019), H. Urbina HU4069 (PUL culture collection; ITS = GenBank MH844000); HU4107 (PUL culture collection; ITS = GenBank MH844024); HU4147 (PUL culture collection; ITS = GenBank MH844045). FINLAND. Hyytiälä Forest station, Mämmilampi site (61.8400, 24.2600, altitude 180 m), surface sterilized root tips of *Pinus sylvestris* (*Pinaceae*), summer 2002 J. Heinonsalo JH144 (HAMBI Culture Collection, ITS = GenBank LK052815), Scots pine forest near Pieksamäki (62.3700, 27.0700, altitude 120 m) surface sterilized root tips of *Pinus sylvestris* (*Pinaceae*), autumn 2008 J. Heinonsalo JH169 (HAMBI Culture Collection, ITS = GenBank LK052826).

##### Notes.

*Semicentenialea rex* sp. nov. represents the first named species of what has previously been known as ‘Clade GS25’ (Tedersoo et al. 2017). There are currently no formally described species closely related to *S. rex* sp. nov. The closest described relatives to *S. rex* sp. nov. belong to other classes within the *Pucciniomycotina (Basidiomycota)*, most likely within the *Classiculomycetes*. Several cultures representing the species were captured from root tips at the sampled location at Ivantjärnsheden Field Station, Jädraås, Sweden. Two additional cultures were obtained from ectomycorrhizal roots of *P. sylvestris* in central Finland. The species corresponds to the UNITE SH1268087.09FU (https://doi.org/10.15156/BIO/SH1268087.09FU) with over 2600 environmental ITS sequences. The species is by far the most frequently recovered USH of the lineage as captured by TH069974.

## Discussion

Previous multi-locus phylogenetic analyses of *Pucciniomycotina* were unable to resolve the backbone topology within the subphylum (Aime et al. 2006; Bauer et al. 2006; Schell et al. 2011; Wang et al. 2015a, 2015b). Using a larger proportion of genomic data and a smaller, strategically reduced taxon sampling, including at least one representative of each known class (with the exception of *Cryptomycocolacomycetes*), our phylogenomic analysis supports some of the deep nodes within the *Pucciniomycotina*, and is congruent with the analyses of Schell et al. (2011), Aime et al. (2014) and Prasanna et al. (2020). Many of the recovered deep nodes still lack strong support, despite sampling from all known classes, supporting the hypothesis that much uncharacterized diversity, especially at the deeper nodes, remains undescribed in *Pucciniomycotina* (Aime et al. 2014). At least three previously unrecognized clades (GS25–27) have been outlined in *Pucciniomycotina* based on eDNA sequencing of the LSU and SSU rDNA regions by Tedersoo et al. (2017). Our analysis provides support for the placement of *Semicentenialomycetes* class. nov., previously known as Clade GS25, within the *Pucciniomycotina*, but does not provide support for the placement of *Semicentenialomycetes* class. nov. as the sister lineage to the rest of the subphylum as hypothesized based on eDNA (Tedersoo et al. 2017). Instead, our analysis reveals that *Classiculomycetes* (represented in our analysis by *Classicula fluitans*) is likely the sister lineage to *Semicentenialomycetes* class. nov. Morphological and ecological data clearly distinguish these two lineages, and *Semicentenialomycetes* is described here as a new class within the *Pucciniomycotina*.

With the help of newly generated genomic and morphological data, we describe the first species of the *Semicentenialomycetes* class. nov. captured in culture, *Semicentenialea rex* sp. nov. Based on eDNA data reported to UNITE database, *S. rex* sp. nov. has a broad geographical distribution and appears to occur at a rather low read abundance when detected. At our study site, *S. rex* sp. nov. has an affinity to mineral soil horizons. It can live in close association with pine roots because it was isolated from pine roots both in Sweden and in Finland. It is possible that *S. rex* sp. nov. lives as a general plant endophyte as indicated by eDNA sequences recovered from leaves (UDB0749737, UDB0753573). In contrast, the currently known species belonging to *Classiculomycetes* are aquatic and associated with leaf litter (Bauer et al. 2003; Aime et al. 2014).

Our morphological examinations of the *S. rex* sp. nov. isolates revealed a mixture of many different developmental structures for which the function remains uncertain. Specifically, we observed three different types of swellings, which were often simultaneously present even on a small part of the culture. Hyphae are primarily dikaryotic with clamp connections formed at some, but not all septa, and at the base of the BLCs. We identified BLCs, but could not observe where karyogamy happens. The TLCs are reminiscent of probasidia/teliospores described from other *Pucciniomycotina*, and could possibly function as resting spores (Aime et al. 2014). A seemingly distinctive character is the observed multinucleate, spore-like cells forming inside of thick-walled TLCs. The TLCs can detach from the hyphae, and the spore-like cells were observed released after disintegration of the TLC cell wall. We did not observe germination of the spore-like cells, and whether they germinate to form multinucleate hyphae or proliferate as yeasts, as well as the ploidy of those cells, remain to be answered in future studies addressing the life cycle of this species. In addition, intercalary swellings were sometimes observed that may act as chlamydospores. It is possible that *S. rex* sp. nov. is unable to complete its entire life cycle in culture, making interpretation difficult, as is the case for many *Cystobasidiomycetes* such as *Bannoa* and *Erythrobasidium*, which appear to form holobasidia in culture, although these have not yet been observed to germinate (Hamamoto et al. 1988, 2002). Future ultrastructural studies with transmission electron microscopy may help to identify meiotic structures and characterize the septal pore, which has been described as an important taxonomic character in basidiomycetes (Bauer et al. 2006). Moreover, time-lapse microscopy will be needed to follow the development of different cell types and to clarify their function.

It remains challenging to estimate the number of species in *Semicentenialomycetes* class. nov. based on available eDNA data. The number of cluster-based USHs may be inflated due to inadequate trimming of the sequence ends, or incomplete ITS1 and ITS2 eDNA amplicon sequences due to truncation, which are known issues in public sequence databases (Nilsson et al. 2017), or errors arising from different sequencing technologies. All sequences annotated as GS25 in UNITE database form three distinct clades in a phylogenetic tree based on alignment of the ITS region, and we conclude that the class contains at least three species, but there is also notable variation in the ITS region within each of these clades. As an example, the clade 1 includes sequences with pairwise similarity of ≥ 94.5%. Further analysis of additional long read data and curated short read data is required to determine whether each of the three clusters represents one or several species. The largest clade is dominated by a frequently observed species hypothesis at 1.5% dissimilarity threshold in the UNITE database (SH1268087.09FU). This USH encompasses sequences from all *S. rex* sp. nov. isolates and the vast majority of eDNA sequences in the class. To better enable cross-study comparison of eDNA amplicon data, we advocate for the use of ASVs rather than other clustering methods. ASVs represent the amplified sequence variants in a sample and are not suitable as species proxies but can be used for building phylogenetic species hypotheses from eDNA amplicon data (Kalsoom Khan et al. 2020). By using ASVs, it is possible to bypass the limitations of clustering methods, such as dataset-dependency or reliance on arbitrarily determined similarity thresholds (Callahan et al. 2017; Cline et al. 2017). Once eDNA from taxa are observed in multiple studies and multiple locations, ASVs can be aligned, and phylogenetic analysis can be used to delineate phylogenetic species hypotheses that can be further tested using associated metadata. Percent sequence similarity is not a meaningful way to capture taxa, instead diagnostic regions in the marker genes can be identified and used for future taxonomic assignments.

Our study highlights the challenges in estimating species richness in eDNA sequence data. First, three *Semicentenialomyces* gen. nov. amplicon sequence variants were detected in eDNA from the study site. Although highly similar, none of these are identical to the rDNA sequences from the different *S. rex* sp. nov. isolates from the same site. The lack of identical ASV matches showcases the limitations in capturing the exact organismal sequences in eDNA amplicon data and is indicative of methodological errors related to PCR and sequencing using different technologies. In addition, it might reflect the intragenomic sequence variation in the ribosomal operon observed in some fungi (Bradshaw et al. 2023; Kijpornyongpan et al. 2024). However, both clustering and phylogenetic analyses demonstrate that the variants represent the same taxa and that it can be distinguished based on eDNA data from other clades in the analysis. It may be irrelevant to search for the exact sequence of a taxon; instead, the power of eDNA amplicon data lies in that multiple observations can be combined and compared, which, when linked to valuable metadata, can inform us about distribution and habitat preference of the studied taxon. Second, we found that our ability to recover a complete ribosomal operon from the genome assembly depended on the assembler used, and that the number of recovered copies varied. We were surprised by the discrepancies in ribosomal operon copy numbers across different genome assemblers and that the genome assembly from the JGI pipeline failed to recover a complete operon, even though the reads were present. However, the challenges of assembling the rDNA operon in genome data were recently highlighted in an extensive study of over 34,000 eukaryote genomes (Krabberød et al. 2025), which showed that the full-length operon could be retrieved only in 34% of genomes. Echoing the conclusions by Krabberød et al. (2025), the errors in operon recovery and sequence variation between different operon copies in our study are probably primarily due to methodological issues during sequencing and assembly, while smaller differences may reflect allelic variation present in our hypothetically heterokaryotic culture. It is also possible that some of the ribosomal reads in our data might belong to the contamination (*Rutstroemiaceae* sp.), and that these were not completely removed in the data cleaning steps, however no matches to *Rutstroemiaceae* were found in our cleaned dataset. Recovering a reliable rDNA operon from a genome assembly is valuable because it provides a link to the extensive metadata on the distribution of that taxon available from eDNA studies.

It was the synergy of characteristics across unrelated studies that allowed us to make the connection between cultured fungus and eDNA sequence data for the case of *S. rex* sp. nov. The incorporation of long-read data by Tedersoo et al. (2017) into a previous global metabarcoding dataset (Tedersoo et al. 2014) allowed these authors to place unidentified eDNA sequences into a phylogenetic framework, while the use of unofficial names like Clade GS25 provided a conduit for cross-study communication about the lineage. Kalsoom Khan et al. (2020) incorporated a simultaneous culture isolation component to their metabarcoding study, which resulted in unidentified fungal cultures that correspond to unidentified fungal sequences detected in eDNA samples. At the time, the unidentified fungal cultures were not of interest to the study at hand (Kalsoom Khan et al. 2020) but were fortunately maintained in the local culture collection, similar to the practice at the Microbial Domain Biological Resource Centre HAMBI, University of Helsinki (Oivanen and Hatakka 2007). The use of unofficial names (e.g., Clade GS25) coupled with an inferred phylogenetic placement of unidentified taxa by Tedersoo et al., (2014, 2017) inspired members of our team to re-visit data collected for the unidentified fungal cultures collected within another study (Kalsoom Khan et al. 2020). Without such attempts, the cultures investigated in the current study may still be unidentified, and Clade GS25 would likely remain without a name according to the code of nomenclature.

## Conclusions

This work highlights the value of coupling eDNA studies with culture-based sampling efforts as demonstrated by the fact that many hitherto unknown fungi can still be captured in culture, and encourages the practice of including phylogenetically informative genes, like 18S or 28S, in metabarcoding studies for the phylogenetic placement of unidentified eDNA sequences. Unofficial names, such as GS25, are useful in the process of detecting and communicating about yet unidentified fungal taxa but provide little additional information beyond knowing if the lineage represents a novel branch at high taxonomic rank. Our study highlights the transient nature of the exact barcode sequences to represent taxa, and we argue that clustering space and phylogenetic relationships should be analyzed before designating novel dark fungal taxa based on eDNA barcode sequences.

Similar to when *Archaeorhizomycetes* was described in 2011, the story of *Semicentenialomycetes* builds on explorative efforts to culture endophytic fungi from coniferous roots and sequence their barcode regions. In both cases it was possible to identify the obtained cultures as novel lineages because previous studies had identified and highlighted these novel lineages based on eDNA long amplicon sequencing and phylogenetic analysis (Schadt et al. 2003; Porter et al. 2008; Tedersoo et al. 2017). We believe that many novel fungal lineages, currently known only from eDNA, can be cultured if researchers are willing to undertake explorative culturing efforts from diverse habitats and obtain barcode sequences from their cultures. Many dark taxa may already be present in culture collections that remain to be barcoded. As a joint effort, such practice could facilitate more discoveries of cultured fungi representing dark taxa.

## Supplementary Material

XML Treatment for Semicentenialomycetes

XML Treatment for Semicenteniales

XML Treatment for Semicentenialaceae

XML Treatment for Semicentenialea

XML Treatment for Semicentenialea rex

## Additional information

### Artificial Intelligence (AI) use

Regarding the use of AI in the preparation of this manuscript, the authors declare the following:

During the preparation of this work, the authors used Microsoft 365 Copilot (2025) exclusively to make minimal linguistic revisions to a few individual sentences. After using this tool, the authors critically reviewed and edited the content as needed and take full responsibility for the content of the published article.

### Adherence to national and international regulations

All the fungal strains used in this study have been legally obtained, respecting the Convention on Biological Diversity (Rio Convention).

### Author ORCIDs

V.T. Nogerius https://orcid.org/0000-0002-8558-3289

M. Sánchez-García https://orcid.org/0000-0002-0635-6281

K. Kluting https://orcid.org/0000-0002-2328-8081

J. Heinonsalo https://orcid.org/0000-0001-8516-1388

M. Ryberg https://orcid.org/0000-0002-6795-4349

M. Toome https://orcid.org/0000-0001-9177-7218

S. Haridas https://orcid.org/0000-0002-0229-0975

S. Mondo https://orcid.org/0000-0001-5797-0647

K. LaButti https://orcid.org/0000-0002-5838-1972

A. Lipzen https://orcid.org/0000-0003-2293-9329

K. Barry https://orcid.org/0000-0002-8999-6785

I.V. Grigoriev https://orcid.org/0000-0002-3136-8903

M.C. Aime https://orcid.org/0000-0001-8742-6685

A. Rosling https://orcid.org/0000-0002-7003-5941

### Data availability

Supporting data such as the custom Julia scripts, alignments and tree files are available in the associated public FigShare project https://doi.org/10.17044/scilifelab.26894242. The genome assemblies and raw reads are available in the NCBI BioProjects (PRJNA218037, PRJNA245617, PRJNA218035, PRJNA218030, PRJNA218039, PRJNA218036, PRJNA218038, PRJNA1466414) and JGI portal (Pucsp_gs25_1, Spoli1, Agahy1, Eryha1, Naose1, Naifl1, Trisp1, Hetpy1).

## Supplementary materials

Supplementary material 1 Supplementary methods, figures, tables, and movie legends Data type pdf https://binary.pensoft.net/file/1704970This dataset is made available under the Open Database License (http://opendatacommons.org/licenses/odbl/1.0/). The Open Database License (ODbL) is a license agreement intended to allow users to freely share, modify, and use this Dataset while maintaining this same freedom for others, provided that the original source and author(s) are credited. Veera Tuovinen Nogerius, Marisol Sánchez-García, Kerri Kluting, Jussi Heinonsalo, Martin Ryberg, Merje Toome, Sajeet Haridas, Stephen Mondo, Kurt LaButti, Matt Nolan, Anna Lipzen, Maxim Koriabine, Diane Bauer, Kerrie Barry, Igor V. Grigoriev, M. Catherine Aime, Anna Rosling

Supplementary material 2 Movie S1 Data type mov Explanation note 3D volume rendering of the z-stack in Fig. 1D. Nuclei stained with propidium iodine (magenta) and cell walls and septa with calcofluor white (cyan). Hypha with clamp connections and nuclei. Different developmental stages of BLCs, probasidia/TLCs and an intercalary swelling, HU4064. https://binary.pensoft.net/file/1704971This dataset is made available under the Open Database License (http://opendatacommons.org/licenses/odbl/1.0/). The Open Database License (ODbL) is a license agreement intended to allow users to freely share, modify, and use this Dataset while maintaining this same freedom for others, provided that the original source and author(s) are credited. Veera Tuovinen Nogerius, Marisol Sánchez-García, Kerri Kluting, Jussi Heinonsalo, Martin Ryberg, Merje Toome, Sajeet Haridas, Stephen Mondo, Kurt LaButti, Matt Nolan, Anna Lipzen, Maxim Koriabine, Diane Bauer, Kerrie Barry, Igor V. Grigoriev, M. Catherine Aime, Anna Rosling

Supplementary material 3 Movie S2 Data type mov Explanation note 3D volume rendering of the z-stack in fig. S2A. Nuclei stained with propidium iodine (magenta) and cell walls and septa with calcofluor white (cyan), HU4064. https://binary.pensoft.net/file/1704972This dataset is made available under the Open Database License (http://opendatacommons.org/licenses/odbl/1.0/). The Open Database License (ODbL) is a license agreement intended to allow users to freely share, modify, and use this Dataset while maintaining this same freedom for others, provided that the original source and author(s) are credited. Veera Tuovinen Nogerius, Marisol Sánchez-García, Kerri Kluting, Jussi Heinonsalo, Martin Ryberg, Merje Toome, Sajeet Haridas, Stephen Mondo, Kurt LaButti, Matt Nolan, Anna Lipzen, Maxim Koriabine, Diane Bauer, Kerrie Barry, Igor V. Grigoriev, M. Catherine Aime, Anna Rosling

Supplementary material 4 Movie S3 Data type mov Explanation note 3D volume rendering of the z-stack in Fig. 3H. Nuclei stained with propidium iodine (magenta) and cell walls and septa with calcofluor white (cyan). Basidium formed from a thick-walled probasidial-like swelling (arrowhead) and forming a second basidium with two nuclei in the obovoid cell and one in the lateral cell, HU4064. https://binary.pensoft.net/file/1704981This dataset is made available under the Open Database License (http://opendatacommons.org/licenses/odbl/1.0/). The Open Database License (ODbL) is a license agreement intended to allow users to freely share, modify, and use this Dataset while maintaining this same freedom for others, provided that the original source and author(s) are credited. Veera Tuovinen Nogerius, Marisol Sánchez-García, Kerri Kluting, Jussi Heinonsalo, Martin Ryberg, Merje Toome, Sajeet Haridas, Stephen Mondo, Kurt LaButti, Matt Nolan, Anna Lipzen, Maxim Koriabine, Diane Bauer, Kerrie Barry, Igor V. Grigoriev, M. Catherine Aime, Anna Rosling

Supplementary material 5 Movie S4 Data type mov Explanation note 3D volume rendering of the z-stack of globose, TLCs with 3 nuclei. Nuclei stained with propidium iodine (magenta) and cell walls and septa with calcofluor white (cyan), HU4064. https://binary.pensoft.net/file/1704973This dataset is made available under the Open Database License (http://opendatacommons.org/licenses/odbl/1.0/). The Open Database License (ODbL) is a license agreement intended to allow users to freely share, modify, and use this Dataset while maintaining this same freedom for others, provided that the original source and author(s) are credited. Veera Tuovinen Nogerius, Marisol Sánchez-García, Kerri Kluting, Jussi Heinonsalo, Martin Ryberg, Merje Toome, Sajeet Haridas, Stephen Mondo, Kurt LaButti, Matt Nolan, Anna Lipzen, Maxim Koriabine, Diane Bauer, Kerrie Barry, Igor V. Grigoriev, M. Catherine Aime, Anna Rosling

Supplementary material 6 Movie S5 Data type mov Explanation note 3D volume rendering of the z-stack in Fig. 4M. Nuclei stained with propidium iodine (magenta) and cell walls and septa with calcofluor white (cyan). Possibly early developmental stages of TLCs with two spots staining strongly with calcofluor white, hypothetical initial stages of the cell-wall of the spore-like cell, HU4147. https://binary.pensoft.net/file/1704974This dataset is made available under the Open Database License (http://opendatacommons.org/licenses/odbl/1.0/). The Open Database License (ODbL) is a license agreement intended to allow users to freely share, modify, and use this Dataset while maintaining this same freedom for others, provided that the original source and author(s) are credited. Veera Tuovinen Nogerius, Marisol Sánchez-García, Kerri Kluting, Jussi Heinonsalo, Martin Ryberg, Merje Toome, Sajeet Haridas, Stephen Mondo, Kurt LaButti, Matt Nolan, Anna Lipzen, Maxim Koriabine, Diane Bauer, Kerrie Barry, Igor V. Grigoriev, M. Catherine Aime, Anna Rosling

Supplementary material 7 Movie S6 Data type mov Explanation note 3D volume rendering of the z-stack in fig. S4G. Germinating TLC. Nuclei stained with propidium iodine (magenta) and cell walls and septa with calcofluor white (cyan), HU4064. https://binary.pensoft.net/file/1704975This dataset is made available under the Open Database License (http://opendatacommons.org/licenses/odbl/1.0/). The Open Database License (ODbL) is a license agreement intended to allow users to freely share, modify, and use this Dataset while maintaining this same freedom for others, provided that the original source and author(s) are credited. Veera Tuovinen Nogerius, Marisol Sánchez-García, Kerri Kluting, Jussi Heinonsalo, Martin Ryberg, Merje Toome, Sajeet Haridas, Stephen Mondo, Kurt LaButti, Matt Nolan, Anna Lipzen, Maxim Koriabine, Diane Bauer, Kerrie Barry, Igor V. Grigoriev, M. Catherine Aime, Anna Rosling
