# Supplementary material for: Another dark taxon comes to light: Semicentenialomycetes, a new class within the Pucciniomycotina (Basidiomycota), and its first described representative, Semicentenialea rex
Source: IMA Fungus. 2026 Jul 1;17:e189848. doi: 10.3897/imafungus.17.189848 (PMC13347111; doi:10.3897/imafungus.17.189848)
Supplement: Supplementary material 1 — Supplementary methods, figures, tables, and movie legends [file imafungus-17-e189848-s001.pdf]

Supplementary File 1

Supplementary Methods

Starting material, DNA and RNA extractions for the additional Pucciniomycotina genomes
A selection of *Pucciniomycotina* cultures were chosen for sequencing to cover all classes: *Agaricostilbum hyphaenes* ATCC MYA-4628, *Erythrobasidium hasegawianum* ATCC 9536, *Heterogastridium pycnidioideum* ATCC MYA-4631, *Classicula fluitans* ATCC 64713, *Naohidea sebacea* CBS 8477, *Phyllozyma linderae* CBS 7893 and *Tritirachium* sp. CBS 265.96. All those isolates were grown on Potato Dextrose Agar (PDA) and five days after inoculation the cells were homogenized by grinding in liquid nitrogen. DNA was extracted following a modified CTAB protocol described in Padamsee et al. (2012). The RNA was extracted with E.Z.N.A. Fungal RNA Kit (Omega Bio-Tek, Norcross, GA, USA) or with RiboPure TM – Yeast Kit (Ambion Inc., Austin, TX, USA). RNA extractions were followed by DNase treatment with TURBO DNase (Ambion Inc.) following the manufacturers' recommendations.

Sequencing, assembly and annotation of the additional Pucciniomycotina genomes
The genomes were sequenced using Illumina. Illumina regular fragment libraries were produced from 100 ng of DNA sheared to 300 bp using the Covaris LE220 (Covaris) and size selected using SPRI beads (Beckman Coulter). The fragments were treated with end-repair, A-tailing, and ligation of Illumina compatible adapters (IDT, Inc) using the KAPA-Illumina library creation kit (KAPA biosystems). For Illumina 4kb long mate-pair CLRS libraries, 5-6 µg of DNA was sheared using the Covaris g-TUBE (Covaris) and gel size selected for 4kb. The sheared DNA was treated with end repair and ligated with biotinylated adapters containing loxP. The adapter ligated DNA fragments were circularized via recombination by a Cre excision reaction (NEB). The circularized DNA templates were then randomly sheared using the Covaris LE220 (Covaris). The sheared fragments were treated with end repair and A-tailing using the KAPA-Illumina library creation kit (KAPA biosystems) followed by immobilization of mate pair fragments on strepavidin beads (Invitrogen). Illumina compatible adapters (IDT, Inc) were ligated to the mate pair fragments, and 10-12 cycles of PCR were used to enrich for the final libraries (KAPA Biosystems).

For the transcriptomes of *Erythrobasidium hasegawianum* and *Naohidea sebacea*, stranded cDNA libraries were generated using the Illumina Truseq Stranded RNA LT kit. mRNA was purified from 1 ug of total RNA using magnetic beads containing poly-T oligos. mRNA was fragmented and reversed transcribed using random hexamers and SSII (Invitrogen) followed by second strand synthesis. The fragmented cDNA was treated with end-pair, A-tailing, adapter ligation, and 8 cycles of PCR. Total RNA was processed using the Illumina TruSeq RNA Sample Preparation Kit (Illumina, Inc. San Diego, CA). All Illumina DNA libraries and the RNA libraries of *E. hasegawianum* and *N. sebacea* were quantified using KAPA Biosystem's next-generation sequencing library qPCR kit (Roche) and run on a Roche LightCycler 480 real-time PCR instrument. The quantified libraries were then multiplexed with other libraries, and the pool of libraries was then prepared for sequencing on the Illumina HiSeq sequencing platform utilizing a TruSeq paired-end cluster kit, v3 or v4, and Illumina's cBot instrument to generate a clustered flow cell for sequencing. Sequencing of the flow cell was performed on the Illumina HiSeq 2000 or 2500 sequencer using HiSeq TruSeq SBS sequencing kits, v3 or v4, following a 2x150 indexed run recipe.

For the rest of the transcriptomes, the polyA RNA was isolated via hybridization to oligo dT magnetic beads, fragmented using heat in the presence of divalent cations for 4 minutes, reverse transcribed using random hexamers to prime first and second strand synthesis. Subsequently cDNA was enzymatically blunted, A-tailed and ligated to indexed Illumina TruSeq adapters.

Following PCR amplification, the samples were titred using a StepOne (Life Technologies) qPCR machine and paired-end sequenced, 2x150 bases, on an Illumina HiSeq2500 using Rapid Chemistry.
Illumina reads filtered for mitochondrial and artifact/process contamination were assembled using All PathsLG release version R46652 or R49403 (Gnerre et al. 2011). For *Agaricostilbum hyphaenes*, Illumina data was first assembled with Velvet (Zerbino and Birney, 2008) to create an in silico long mate-pair library with insert 3000 +/- 300 bp and another with insert 6000 +- 10 bp. These two long-mate-pair libraries were then assembled with the original Illumina library with AllPathsLG release version R47710.

Raw RNA-Seq reads were filtered and trimmed for quality and contamination. Using BBDuk (https://sourceforge.net/projects/bbmap/), raw reads were evaluated for artifact sequence by kmer matching (kmer=25), allowing 1 mismatch and detected artifact was trimmed from the 3' end of the reads. RNA spike-in reads, PhiX reads and reads containing any Ns were removed. Quality trimming was performed using the phred trimming method set at Q6. Finally, following trimming, reads under the length threshold were removed (minimum length 25 bases or 1/3 of the original read length - whichever is longer). Filtered reads were assembled into consensus sequences using Rnnotator v. 3.4.0) (Martin et al. 2010).

All genomes were annotated using the JGI Annotation pipeline and made available from JGI MycoCosm (Grigoriev et al. 2014). Genome assemblies and annotations were also deposited at GenBank (Suppl. file 1: Table S3).

Supplementary figures

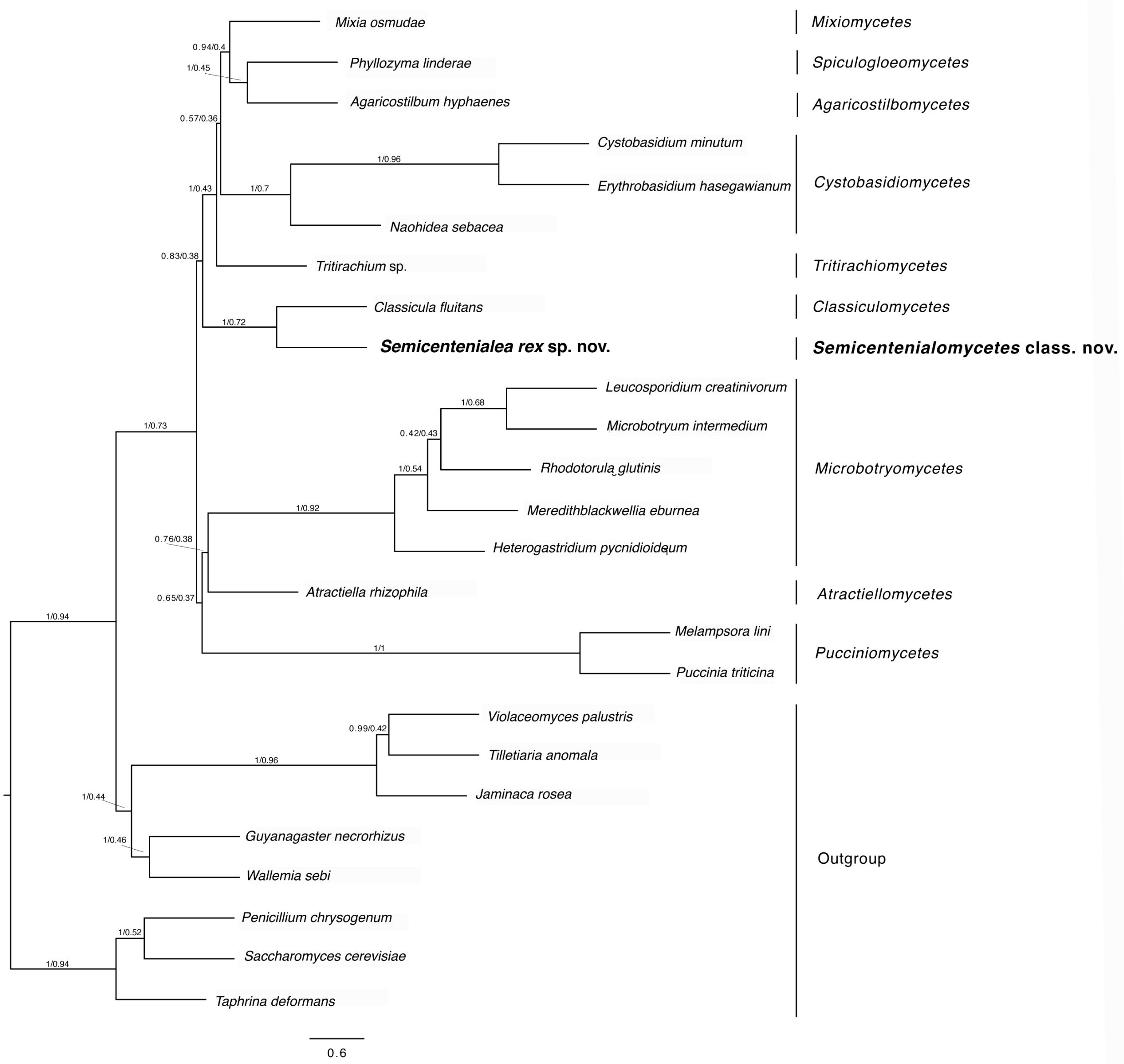

Figure S1. Coalescent-based species tree estimate from the ASTRAL analysis used in Fig. 1. Support values for each node are reported as local posterior probabilities (LPP) and quartet scores from the ASTRAL analysis (LPP/quartet score).

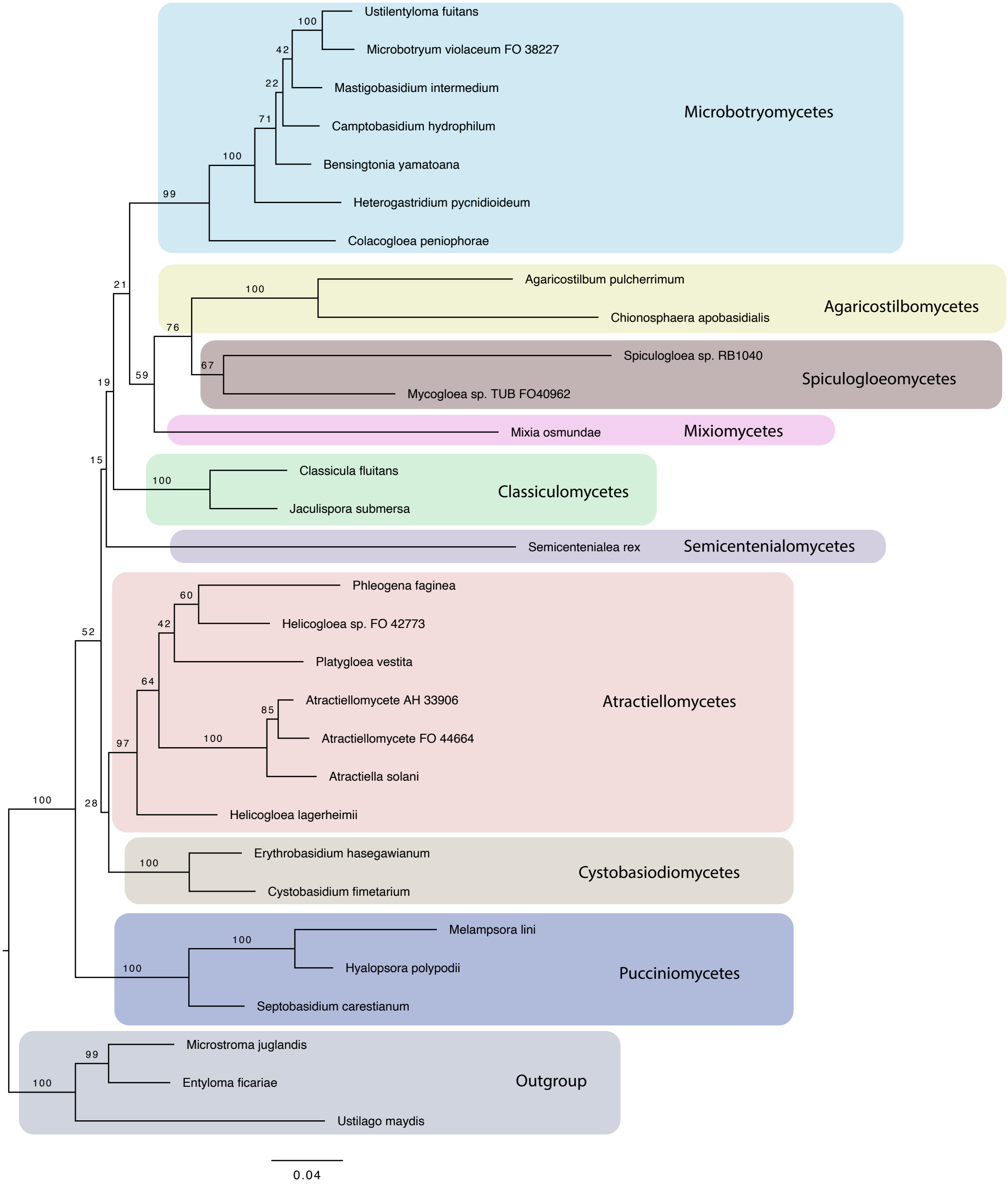

Figure S2. Maximum Likelihood (ML) phylogenetic tree of the *Pucciniomycotina* based on a concatenated analysis of SSU and LSU rDNA sequences. Numbers on the branches indicate bootstrap support. GenBank accession numbers are indicated in Suppl. file 1: Table S4.

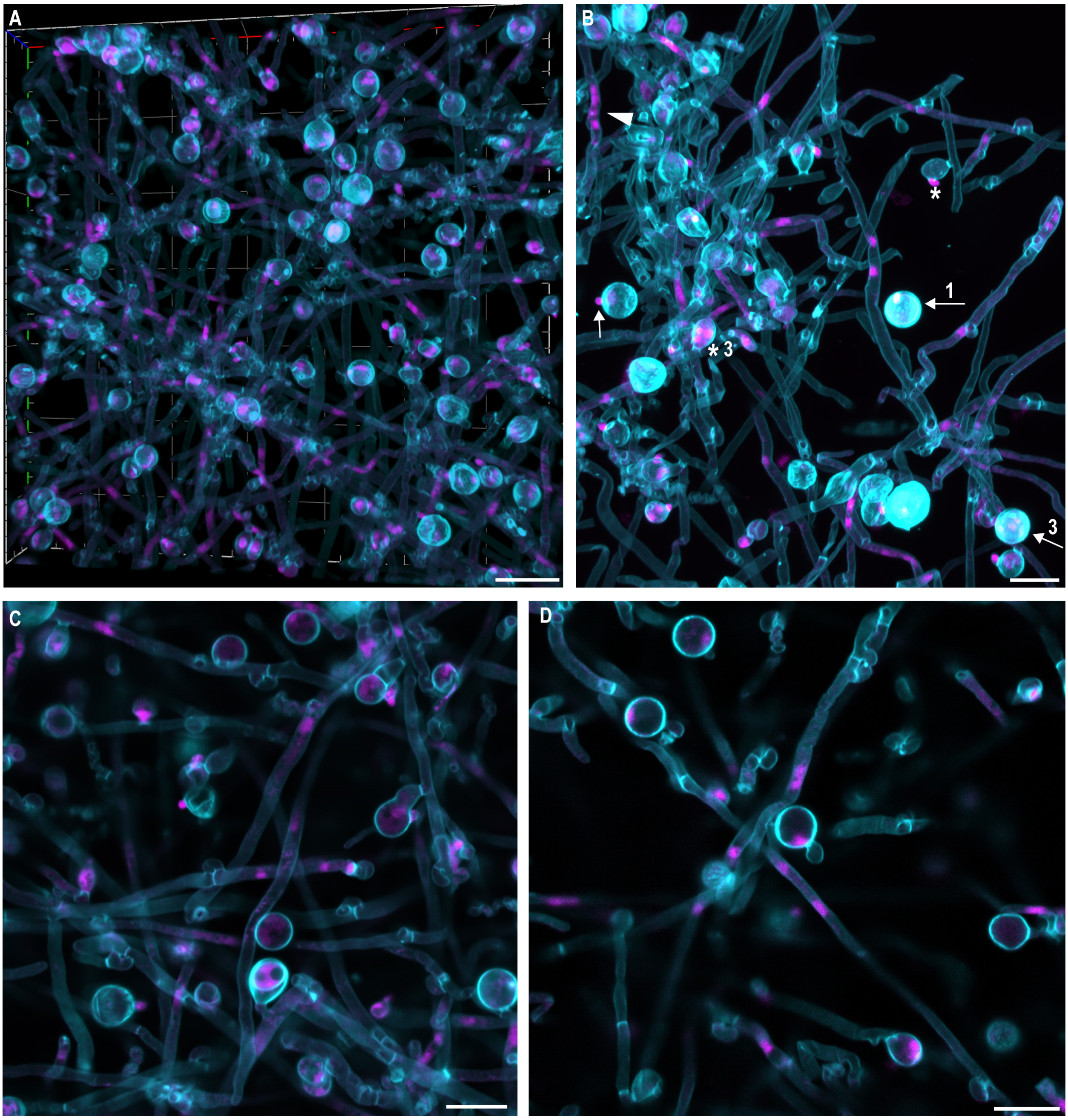

Figure S3. Overview of mycelia. Nuclei stained with propidium iodine (magenta) and cell walls and septa with calcofluor white (cyan), maximum intensity projection. **A** Teliospore-like cells and basidia-like cells (herein TLC and BLC), 3D volume renderings of a z-stack. Rotating 3D rendering of A as Suppl. file 3: Movie S2. **B** Hypha with four nuclei (arrowhead), TLC with a bud with one nucleus (arrow), detached TLC with 1 nucleus (arrow +1), and three nuclei (arrow +3), and BLC with only one nucleus in the lateral cell (asterisk), 3D volume renderings of a z-stack. **C** Dikaryotic hyphae with BLCs in different developmental stages, one optical section. **D** Dikaryotic hyphae with clamps and TLCs budding, one optical section. Isolate HU4064. Scale bars 20 µm (**A**); 10 µm (**B**–**D**).

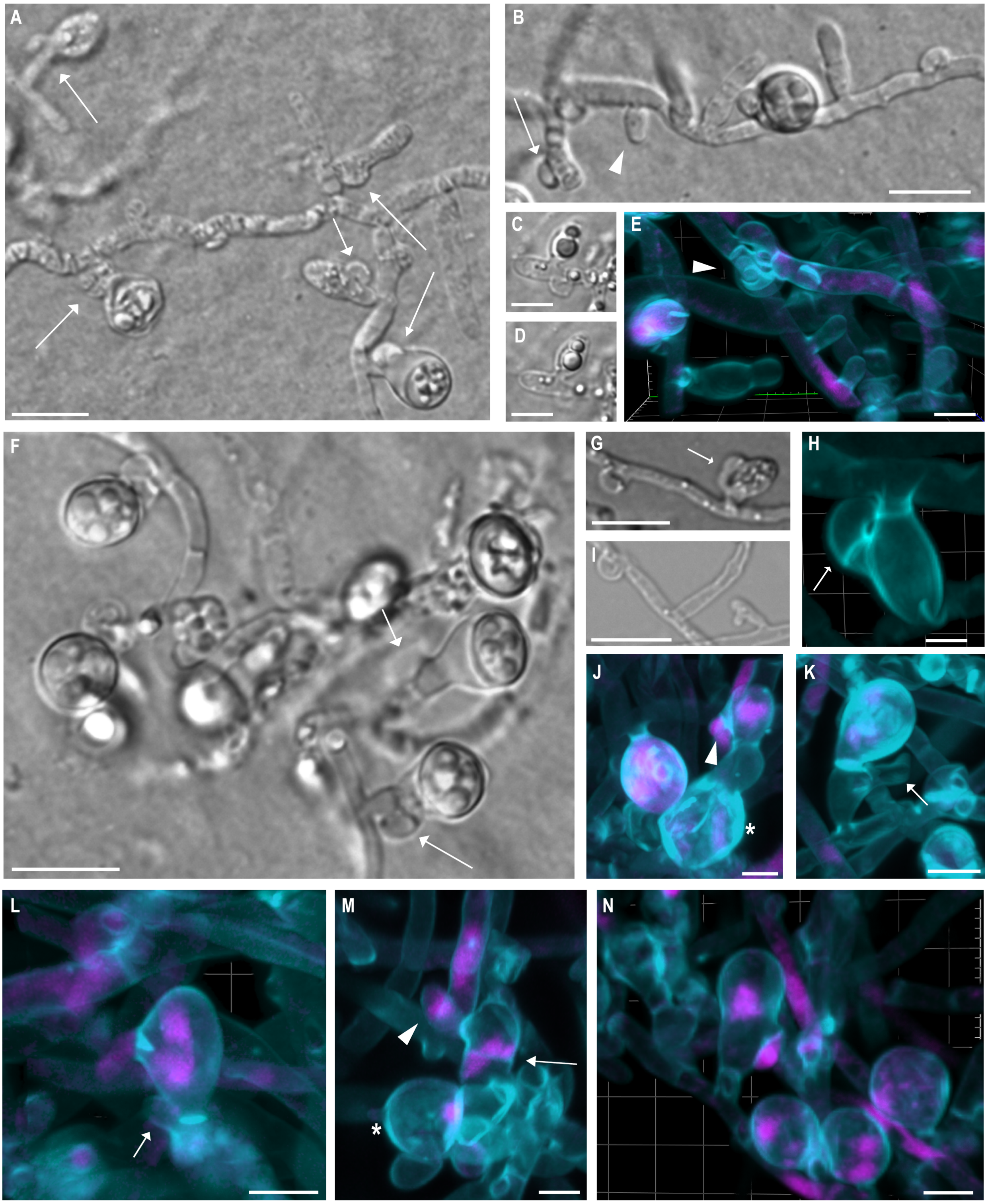

Figure S4. Variation in the morphology of the BLCs. **E**, **H**, **J**–**N**: 3D volume renderings of z-stacks, nuclei stained with propidium iodine (magenta) and cell walls and septa with calcofluor white (cyan). **A–B, F–G** Different developmental stages of BLCs with basal clamp (arrow), yeast budding from hypha in B (arrowhead). **C–D** The same BLC with large lateral cell from two different depths of view. **E** Hyphae anastomosing (arrowhead). **H** Clamp anastomosing back to the generative hypha (arrow). **I** Small BLC. **J** BLC with one nucleus in the lateral cell (arrowhead) developed from a TLC (asterisk). **K–L** Lateral cell arising from the basidium stalk (arrow). **M** BLC with one lateral cell (arrowhead) and a ring with strong calcofluor staining (arrow) dividing the obovoid cell into two compartments with one nucleus on one side and two on the other. A bud-like appendage (asterisk) forming on a TLC. **N** BLC with a short stalk, with two nuclei in the obovoid cell and one in the lateral cell. Isolates HU4107 (**A–F**); HU4064 (**G**, **J–N**); HU4068 (**H**), JH144 (**I**). Scale bars 10 µm (**A–C**, **E–G**, **I**); 4 µm (**D**, **K**, **N**); 2 µm (**H**) ; 3 µm (**J**, **L**, **M**).

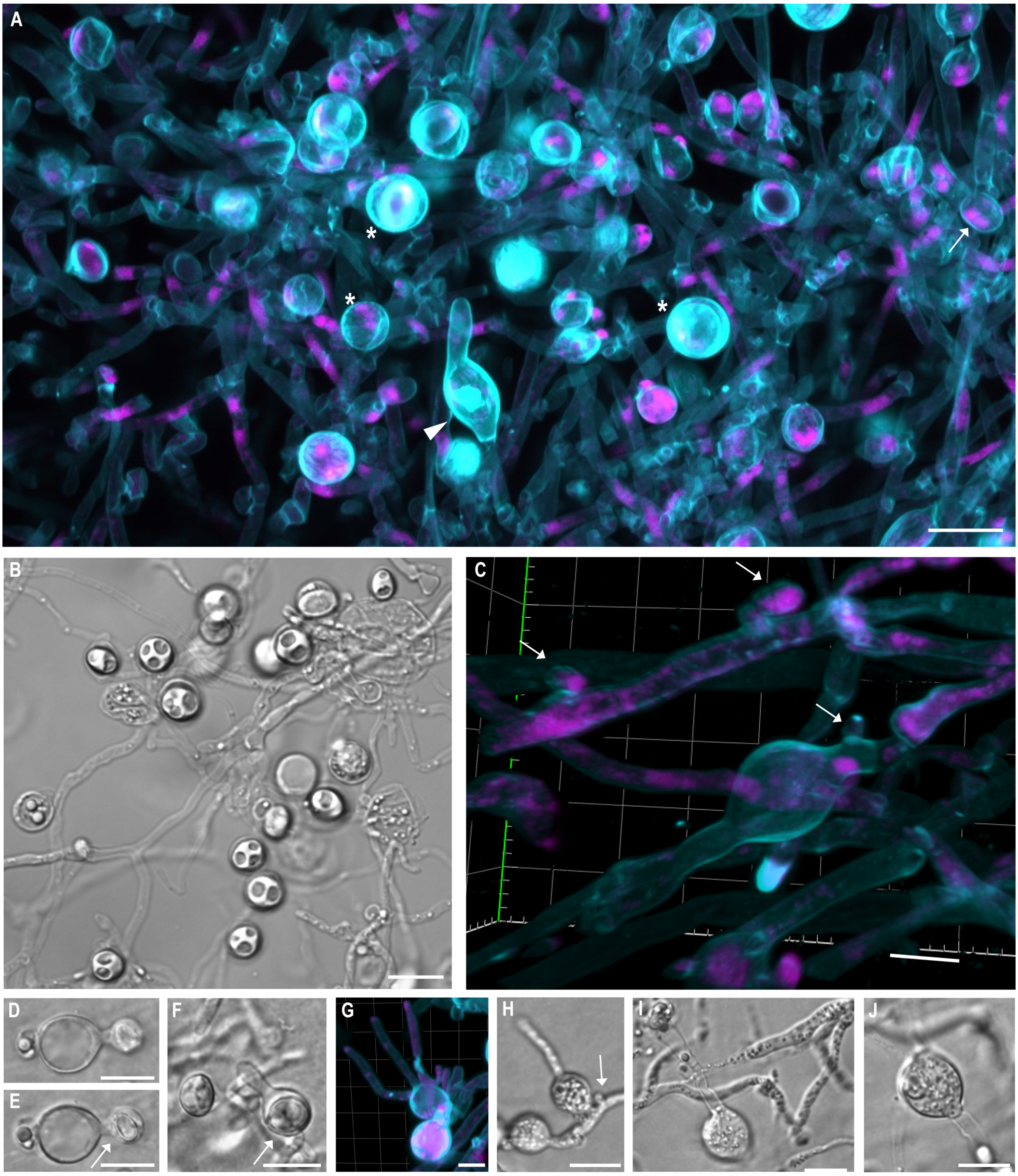

Figure S5. Variation in intercalary swellings and TLCs in A, C and G. **A** Overview of dikaryotic mycelium with many thick-walled TLCs (asterisk) with a developing "spore" inside, an intercalary swelling with septa at one end (arrowhead), and binucleate "spore" (arrow) released from a TLC. **B** TLCs are compartmentalized in brightfield microscopy. **C** Hypha with lateral yeast cells budding (arrow). **D–E** A large TLC connected to a smaller TLC (arrow). **F–G** Germinating TLC. Rotating 3D rendering of G as Suppl. file 7: Movie S6. **H** Germinating TLC attached to a promycelium, with a bud-like appendage at the base (arrow). **I–J**: An intercalary swelling, two different depths of view. Isolates HU4107 (**D–F**); HU4064 (**C**, **G**, **I–J**); JH144 (**B**, **H**). Scale bars 10 µm (**A–B**, **D–F**, **H–J**); 5 µm (**C**); 4 µm (**G**).

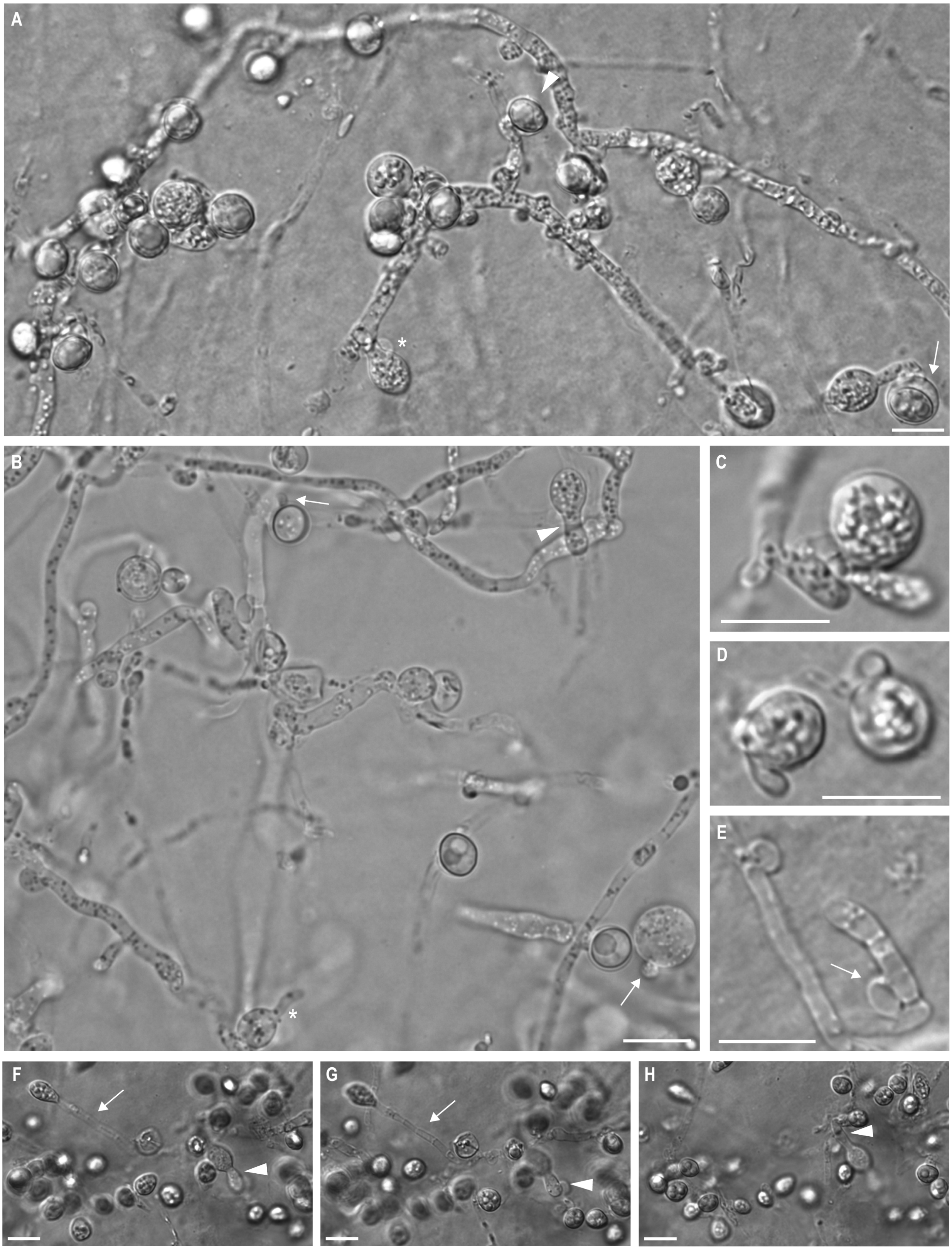

Figure S6. Promycelium and TLC. A TLC with a spore-like cell inside (arrow), spore-like cell (arrowhead), BLC with a clamp (asterisk). **B** Budding TLCs (arrow), TLC germinating to promycelium (arrowhead), budding TLC attached to hypha (asterisk). **C** Promycelium and TLC. **D** Budding TLCs. **E** Hyphae with a clamp and budding lateral cell (arrow). **F–H** TLC germinating forming septate hyaline hyphae (arrow), and TLC germinating to two directions (arrowhead). Different depths of view. Isolates HU4107 (**D–F**); HU4064 (**C**, **G**, **I–J**); JH144 (**B**, **H**). Scale bars 10 µm.

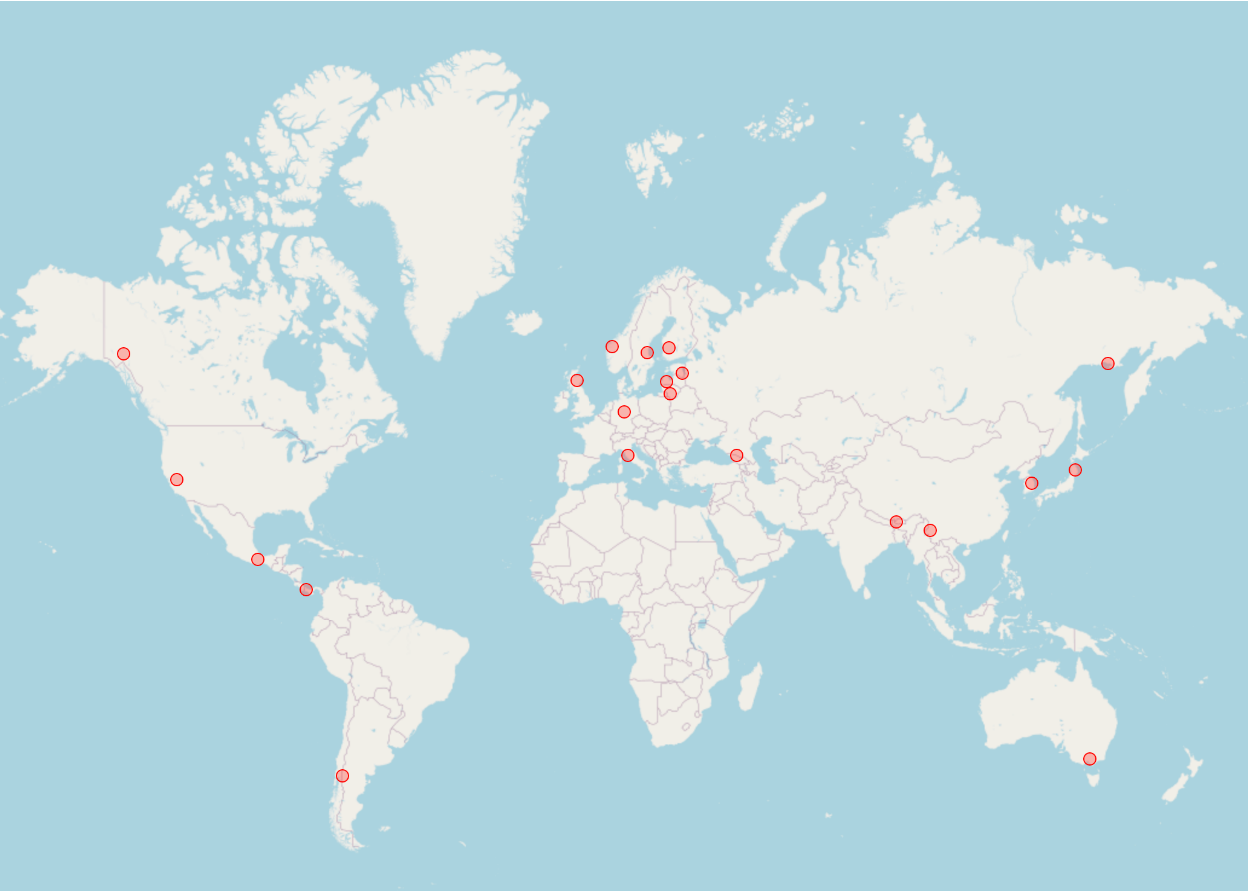

Figure S7. Global distribution of *S. rex* sp. nov. as estimated by the metadata associated with detection of eDNA ITS amplicon sequences clustered in the UNITE species hypothesis SH1268087. https://doi.plutof.ut.ee/doi/10.15156/BIO/SH1268087.09FU.

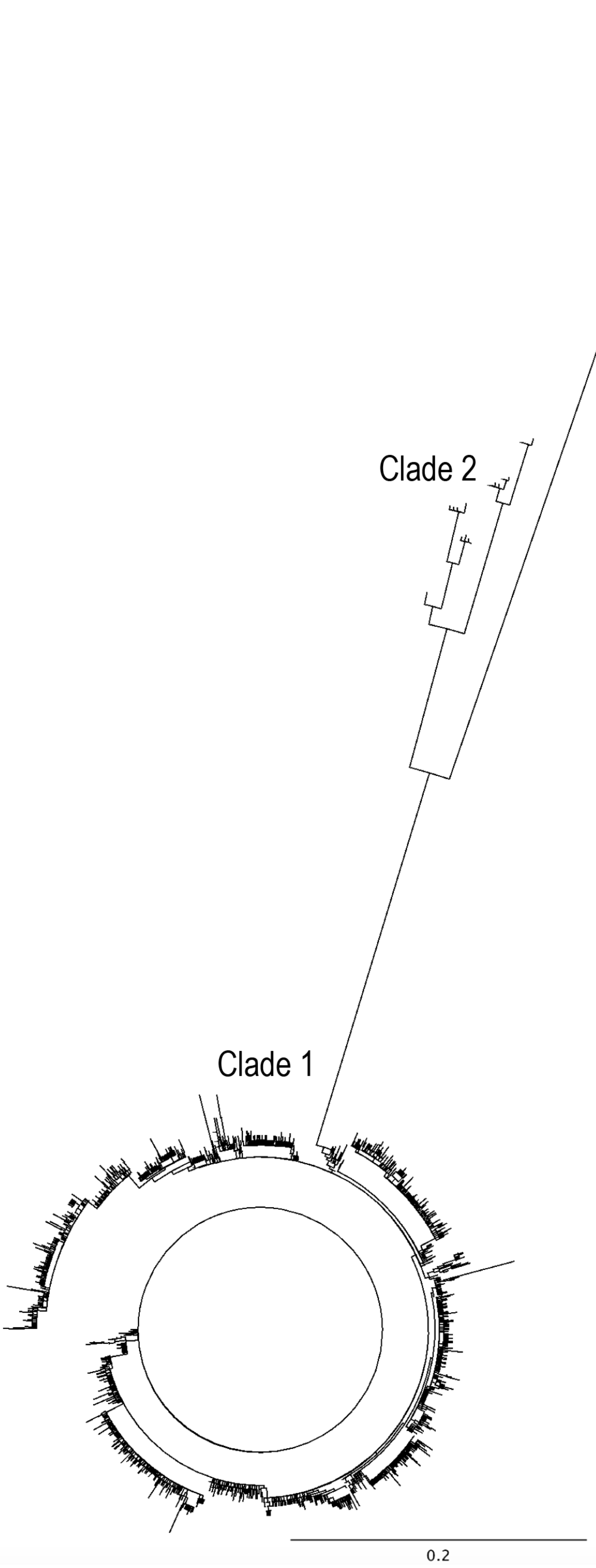

Figure S8. Mid-point rooted ML tree of all 1426 unique sequences annotated as GS25 in the UNITE database version 9. Of the sequences, 98% form a clade dominated by *S. rex* (SH1268087). Remaining sequences form two loose clades (2 and 3).

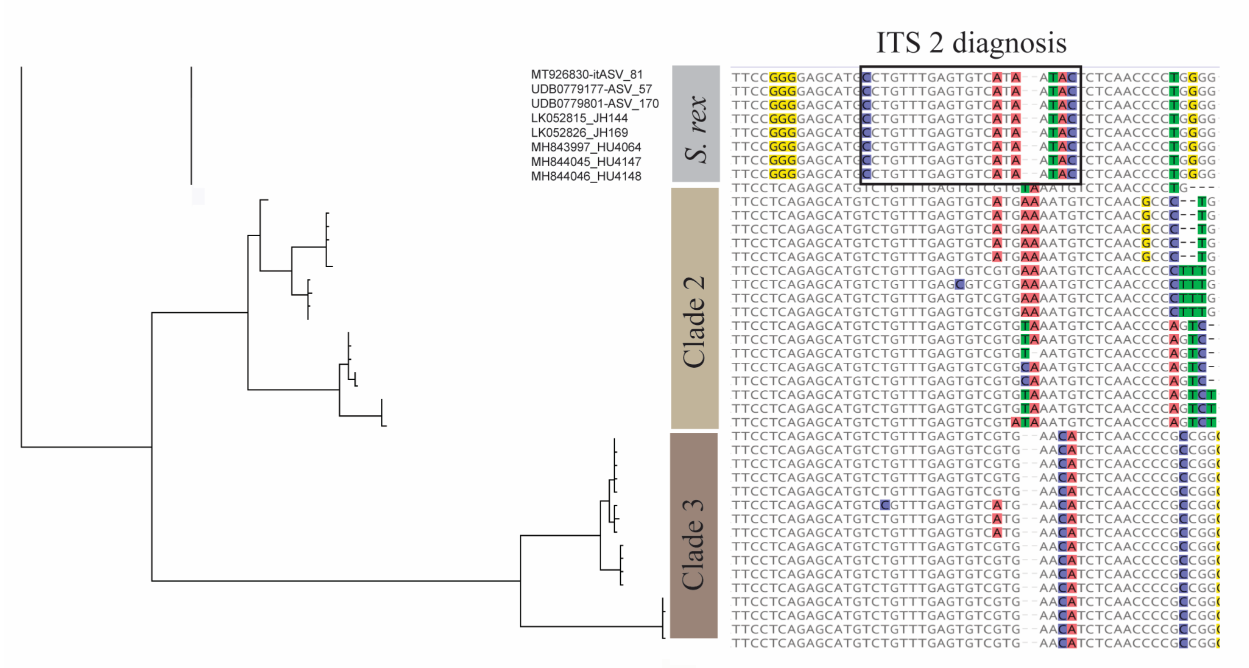

Figure S9. Diagnostic ITS2 sequence for cultures of *S. rex.* A 21 bp diagnostic sequence in the ITS2 region corresponding to bases 181–201 in MH843997 can be used to distinguishing sequences representing *S. rex* from those of the other yet undescribed taxa in the class. By allowing for 1 bp mismatch all unique sequences in clade 1 match the region and can be distinguished from all unique sequences in clades 2 and 3. The region is visualized next to a schematic illustration of the phylogenetic relation between the three clades in Suppl. file1: Fig. S7.

Supplementary Tables

Table S1. Fungal isolates from Ivantjänsheden Field Station assigned to Clade GS25 in Kalsoom Khan et al (2020) and from Finland, with GenBank accession number, status of the culture and the sample origin of the isolate.

Isolate	GenBank Acc nr.	Sequenced region	Extant culture	Sample of isolation
HU4006	MH843967	ITS2-LSU		17I-B-27-1, Kalsoom Khan et al (2020)
HU4060	MH843995	ITS2-LSU		17I-B-15-5, Kalsoom Khan et al (2020)
HU4063	MH843996	ITS2-LSU		17I-B-15-4, Kalsoom Khan et al (2020)
HU4064	MH843997	ITS-LSU	Rosling lab, Uppsala University, Sweden; CBS 150710; PUL culture collection	not recorded
HU4068	PQ270019	ITS-LSU	Rosling lab, Uppsala University, Sweden	4IF-B-8-2, Kalsoom Khan et al (2020)
HU4069	MH844000	ITS2-LSU	Rosling lab, Uppsala University, Sweden	17I-B-15-1, Kalsoom Khan et al (2020)
HU4107	MH844024	LSU	Rosling lab, Uppsala University, Sweden	4IF-B-23-1, Kalsoom Khan et al (2020)
HU4120	MH844033	ITS2-LSU		not recorded
HU4147	MH844045	ITS	Rosling lab, Uppsala University, Sweden	not recorded
HU4148	MH844046	ITS		not recorded
HU4150	MH844048	LSU		not recorded
JH144	LK052815	ITS	HAMBI Culture Collection, University of Helsinki, Finland	N/A
JH169	LK052826	ITS	HAMBI Culture Collection, University of Helsinki, Finland	N/A

Table S2. Assembly statistics for the different genome assemblers and their performance in assembling the ribosomal operon (full and ITS region).

Assembly*	# contigs	Largest contig	Total length (bp)	GC (%)	N50	N75	L50	L75	Busco (total)	Busco (S)	Busco (D)	Busco (F)	ITS region and % similarity to MH843997	Full ribosomal operon
JGI	405	1232725	54259369	53.79	289506	139999	55	123	95,2	29,6	65,6	0,8	1 copy of 703 bp 98% similarity	2 partial copies of ca 2700 bp
Canu	1362	4301668	88868746	50.81	272332	38493	51	278	97	39,3	57,7	0,6	34 copies of ca 1014 bp 99% similarity	30 copies of ca 5700 bp
Flye	479	4301918	67940192	49.93	567724	177750	25	78	96,5	64,5	32	0,6	1 copy of 1014 bp 99% similarity	1 copy of ca 5500 bp
Flye (hifi)	164	4301793	112044026	51.45	1015173	735514	32	65	97,6	4,4	93,2	0,6	15 copies of ca 1014 bp 99% similarity	10 copies of ca 5700 bp
Hifiasm	106	4301791	87748252	50.75	1523022	869692	21	41	97,3	20,4	76,9	0,6	50 copies of ca 1014 bp 99% similarity	45 copies of ca 5700 bp

*The following genome assemblers were used: canu (v.2.2) with standard settings, Flye (v.2.9.5) with standard settings and with the hifi setting, and hifiasm (v. 0.20.0-r639) with standard settings.

Table S3. Genomes used in phylogenomic analyses. NCBI Bioproject/Genome assembly accession number are given for the newly generated genomes. *Unpublished genome, used with permission.

***Pucciniomycotina* class**	**Species**	**JGI portal id**	**NCBI Bioproject/ Accession**	**Original publication**
Spiguloclœomycetes	*Phyllozyma linderae* CBS 7893	Spoli1	PRJNA218037/ JBYDIZ000000000	This study
Agaricostilbomycetes	*Agaricostilbum hyphaenes* ATCC MYA-4628	Agahy1	PRJNA245617/ JBYDIU000000000	This study
Atractiellomycetes	*Atractiella rhizophila*	Atrsp2		Gregory Bonito*
Cystobasidiomycetes	*Cystobasidium minutum* MCA 4210	Rhomi1		Camiolo et al., 2019
	Erythrobasidium hasegawianum ATCC 9536	Eryha1	PRJNA218035/ JBYDIV000000000	This study
	Naohidea sebacea CBS 8477 (P95)	Naose1	PRJNA218030/ JBYDIY000000000	This study
Mixiomycetes	*Mixia osmundae* IAM 14324	Mixos1		Toome et al., 2014a
Classiculomycetes	*Classicula fluitans* ATCC 64713	Naifl1	PRJNA218039/ JBYDIX000000000	This study
Tritirachiomycetes	*Tritirachium* sp. CBS 265.96	Trisp1	PRJNA218036/ JBYDJA000000000	This study
Microbotryomycetes	*Heterogastridium pycnidioideum* ATCC MYA-4631 (P27)	Hetpy1	PRJNA218038/ JBYDIW000000000	This study
	Rhodotorula glutinis ATCC 204091	Rhoglu91_1		Paul et al., 2014
	Leucosporidium creatinivorum UCDFST 62-1032	Leucr1		Mondo et al., 2017
	Microbotryum intermedium 1389 BM 12 12	Micin1		Branco et al., 2017
	Meredithblackwellia eburnea MCA 4105	Mereb1		Mondo et al., 2025
Pucciniomycetes	*Melampsora lini* CH5	Melli1		Nemri et al., 2014

	Puccinia triticina 1-1 BBBD Race 1	Puctr1		Cuomo et al., 2017
Semicentenialomycetes class. nov.	*Semicentenialea rex* sp. nov.	Pucsp_gs25_1	PRJNA1466414/ JBZUJS000000000	This study
**Outgroup taxa**				
Basidiomycota: Ustilaginomycotina	*Tilletiaria anomala* UBC 951	Tilan2		Toome et al., 2014b
	Violaceomyces palustris SA 807	Ustsp1		Kijpornyongpan et al., 2018; Albu et al., 2015
	Jaminaea sp. MCA 5214	Jamsp1		Kijpornyongpan et al., 2018
Basidiomycota: Agaricomycotina	*Wallemia sebi* CBS 633.66	Walse1		Padamsee et al., 2012
	Guyanagaster necrorhizus MCA 3950	Guyne1		Koch et al., 2021
Ascomycota: Saccharomycotina	*Saccharomyces cerevisiae* M3837	SacceM3837_1		Brown et al., 2013
Ascomycota: Taphrinomycotina	*Taphrina deformans*	Tapde1_1		Cissé et al., 2013
Ascomycota: *Pezizomycotina*	*Penicillium chrysogenum* 4088766	Pench1		de Vries et al., 2017

Table S4. Accession numbers of the sequences used for the phylogenetic tree of Pucciniomycotina in Suppl. file 1: Figure S2.

Species name	LSU	SSU
Classicula fluitans	AY512838	AY124478
Agaricostilbum pulcherrimum	L20277	U40809
Chionosphaera apobasidialis	AF393470	U77662
Spiculogloea sp. RB1040	Y512885	DQ198784
Mycogloea sp. TUB FO40962	Y512868	DQ198791
Bensingtonia yamatoana	Y512834	D38239
Mastigobasidium intermedium	Y512859	D38235
Ustilentyloma fluitans	AF009882	AY124481
Microbotryum violaceum FO 38227	AF009866	DQ198782
Camptobasidium hydrophilum	Y512837	DQ198783
Colacogloea peniophorae	Y512839	DQ363320
Heterogastridium pycnidioideum	Y512851	DQ198785
Jaculispora submersa	Y512853	AY124477
Mixia osmundae	AY512867	D14163
Platygloea vestita	Y512872	AY124480
Helicogloea sp. FO 42773	Y512847	DQ198793
Phleogena faginea	Y512869	DQ198798
Atractiellomycete AH 33906	DQ363323	DQ198796
Atractiellomycete FO 44664	DQ363322	DQ198795
Atractiella solani	Y512831	DQ198797
Helicogloea lagerheimii	Y512849	DQ198794
Septobasidium carestianum	L20281	DQ198787
Melampsora lini	L20283	AY125396
Hyalopsora polypodii	Y512852	AB011015
Erythrobasidium hasegawianum	AF131058	D12803
Cystobasidium fimetarium	Y512843	AY124479
Ustilago maydis	L20287	X62396
Microstroma juglandis	AF009867	DQ363313
Entyloma ficariae	AJ235295	DQ198790

Table S5. Sequence similarity in % across the ITS2 region of cultures and ASV sequences.

	LK052815 **JH_144**	LK052826 **JH_169**	MH84399 7**HU4064**	UDB0779177 **ASV_57**	MT926830 **itASV_481**	UDB077981 **ASV_170**	MH844045 **HU4147**	MH844046 **HU4148**
LK052815 **JH144**		99.3	99.6	99.6	96.7	98.1	100	100
LK052826 **JH169**	99.3		99.6	99.6	97.5	98.9	99.1	99.1
MH843997 **HU4064**	99.6	99.6		99.0	97.4	98.7	99.5	99.5
UDB0779177 **ASV_57**	99.6	99.6	99.0		97.1	98.4	99.5	99.5
MT926830 **itASV_481**	96.7	97.5	97.4	97.1		98.7	96.2	96.3
UDB0779801 **ASV_170**	98.1	98.9	98.7	98.4	98.7		97.7	97.7
MH844045 **HU4147**	100	99.1	99.5	99.5	96.3	97.7		100
MH844046 **HU4148**	100	99.1	99.5	99.5	96.3	97.7	100	

Table S6. The taxon hypothesis TH069974 (GS25 ord. Incertae sedis) includes 19 species hypotheses (SH) at the 1.5% dissimilarity threshold in the version 9 of the UNITE database.

UNITE SH	No. of seq.	Detected	SH DOI	Clade
SH1268087.09FU	2 681	Global	https://dx.doi.org/10.15156/BIO/SH1268087.09FU	1
SH1268090.09FU	1	Norway	https://dx.doi.org/10.15156/BIO/SH1268090.09FU	1
SH1268089.09FU	1	Australia	https://dx.doi.org/10.15156/BIO/SH1268089.09FU	1
SH1268094.09FU	1	Russia	https://dx.doi.org/10.15156/BIO/SH1268094.09FU	1
SH1268088.09FU	1	Australia	https://dx.doi.org/10.15156/BIO/SH1268088.09FU	1
SH1268092.09FU	1	Canada	https://dx.doi.org/10.15156/BIO/SH1268092.09FU	1
SH1268091.09FU	1	Finland	https://dx.doi.org/10.15156/BIO/SH1268091.09FU	1
SH1268086.09FU	1	Finland	https://dx.doi.org/10.15156/BIO/SH1268086.09FU	1
SH1268095.09FU	1	Estonia	https://dx.doi.org/10.15156/BIO/SH1268095.09FU	1
SH1268097.09FU	1	Estonia	https://dx.doi.org/10.15156/BIO/SH1268097.09FU	1
SH1268098.09FU	1	Estonia	https://dx.doi.org/10.15156/BIO/SH1268098.09FU	1
SH0896873.09FU	8	Colombia & Guatemala	https://dx.doi.org/10.15156/BIO/SH0896873.09FU	2
SH0896871.09FU	6	Fiji	https://dx.doi.org/10.15156/BIO/SH0896871.09FU	2
SH0896870.09FU	5	Fiji	https://dx.doi.org/10.15156/BIO/SH0896870.09FU	2
SH0896872.09FU	3	Tanzania	https://dx.doi.org/10.15156/BIO/SH0896872.09FU	2
SH0896869.09FU	2	Papua New Guinea	https://dx.doi.org/10.15156/BIO/SH0896869.09FU	2
SH0891076.09FU	10	Dominica & Costa Rica	https://dx.doi.org/10.15156/BIO/SH0891076.09FU	3
SH0891078.09FU	6	China	https://dx.doi.org/10.15156/BIO/SH0891078.09FU	3
SH0891077.09FU	4	Mexico	https://dx.doi.org/10.15156/BIO/SH0891077.09FU	3

Supplementary movie legends

Movie S1. 3D volume rendering of the z-stack in Fig. 1D. Nuclei stained with propidium iodine (magenta) and cell walls and septa with calcofluor white (cyan). Hypha with clamp connections and nuclei. Different developmental stages of BLCs, TLCs and an intercalary swelling, HU4064.

Movie S2. 3D volume rendering of the z-stack in Fig. S3A. Nuclei stained with propidium iodine (magenta) and cell walls and septa with calcofluor white (cyan). TLCs and BLCs., HU4064.

Movie S3.

3D volume rendering of the z-stack in Fig. 3H. Nuclei stained with propidium iodine (magenta) and cell walls and septa with calcofluor white (cyan). BLC formed from a thick-walled probasidial-like swelling (arrowhead) and forming a second BLC with two nuclei in the obovoid cell and one in the lateral cell, HU4064.

Movie S4.

3D volume rendering of the z-stack of globose, TLCs with 3 nuclei. Nuclei stained with propidium iodine (magenta) and cell walls and septa with calcofluor white (cyan), HU4064.

Movie S5. 3D volume rendering of the z-stack in Fig. 4M. Nuclei stained with propidium iodine (magenta) and cell walls and septa with calcofluor white (cyan). Possibly early developmental stages of TLCs with two spots staining strongly with calcofluor white, hypothetical initial stages of the cell-wall of the spore-like cell, HU4147.

Movie S6. 3D volume rendering of the z-stack in Fig. S5G. Germinating TLC. Nuclei stained with propidium iodine (magenta) and cell walls and septa with calcofluor white (cyan), HU4064.

Supplementary references

Albu S, Toome M, Aime MC (2015) Violaceomyces palustris gen. et sp. nov. and a new monotypic lineage, Violaceomycetales ord. nov. in Ustilaginomycetes. Mycologia 107: 1193–1204. https://doi.org/10.3852/14-260

Branco S, Badouin H, Rodríguez de la Vega RC, Gouzy J, Carpentier F, Aguileta G, Siguenza S, Brandenburg J-T, Coelho MA, Hood ME, Giraud T (2017) Evolutionary strata on young mating-type chromosomes despite the lack of sexual antagonism. Proceedings of the National Academy of Sciences 114: 7067–7072. https://doi.org/10.1073/pnas.1701658114

Brown NA, de Castro PA, de Castro Pimentel Figueiredo B, Savoldi M, Buckeridge MS, Lopes ML, de Lima Paullilo SC, Borges EP, Amorim HV, Goldman MHS, Bonatto D, Malavazi I, Goldman GH (2013) Transcriptional profiling of Brazilian Saccharomyces cerevisiae strains selected for semi-continuous fermentation of sugarcane must. FEMS Yeast Research 13: 277–290. https://doi.org/10.1111/1567-1364.12031

Camiolo S, Toome-Heller M, Aime MC, Haridas S, Grigoriev IV, Porceddu A, Mannazzu I (2019) An analysis of codon bias in six red yeast species. Yeast 36: 53–64. https://doi.org/10.1002/yea.3359

Cissé OH, Almeida JMGCF, Fonseca A, Kumar AA, Salojärvi J, Overmyer K, Hauser PM, Pagni M (2013) Genome sequencing of the plant pathogen Taphrina deformans, the causal agent of peach leaf curl. mBio 4: e00055-00013. https://doi.org/10.1128/mBio.00055-13

Cuomo CA, Bakkeren G, Khalil HB, Panwar V, Joly D, Linning R, Sakthikumar S, Song X, Adiconis X, Fan L, Goldberg JM, Levin JZ, Young S, Zeng Q, Anikster Y, Bruce M, Wang M, Yin C, McCallum B, Szabo LJ, Hulbert S, Chen X, Fellers JP (2017) Comparative Analysis Highlights Variable Genome Content of Wheat Rusts and Divergence of the Mating Loci. G3 (Bethesda, Md.) 7: 361–376. https://doi.org/10.1534/g3.116.032797

Kijpornyongpan T, Mondo SJ, Barry K, Sandor L, Lee J, Lipzen A, Pangilinan J, LaButti K, Hainaut M, Henrissat B, Grigoriev IV, Spatafora JW, Aime MC (2018) Broad Genomic Sampling Reveals a Smut Pathogenic Ancestry of the Fungal Clade Ustilaginomycotina. Molecular Biology and Evolution 35: 1840–1854. https://doi.org/10.1093/molbev/msy072

de Vries RP, Riley R, Wiebenga A, Aguilar-Osorio G, Amillis S, Uchima CA, Anderluh G, Asadollahi M, Askin M, Barry K, Battaglia E, Bayram Ö, Benocci T, Braus-Stromeyer SA, Caldana C, Cánovas D, Cerqueira GC, Chen F, Chen W, Choi C, Clum A, dos Santos RAC, de Lima Damásio AR, Diallinas G, Emri T, Fekete E, Flipphi M, Freyberg S, Gallo A, Gournas C, Habgood R, Hainaut M, Harispe ML, Henrissat B, Hildén KS, Hope R, Hossain A, Karabika E, Karaffa L, Karányi Z, Kraševec N, Kuo A, Kusch H, LaButti K, Lagendijk EL, Lapidus A,

Levasseur A, Lindquist E, Lipzen A, Logrieco AF, MacCabe A, Mäkelä MR, Malavazi I, Melin P, Meyer V, Mielnichuk N, Miskei M, Molnár ÁP, Mulé G, Ngan CY, Orejas M, Orosz E, Ouedraogo JP, Overkamp KM, Park H-S, Perrone G, Piumi F, Punt PJ, Ram AFJ, Ramón A, Rauscher S, Record E, Riaño-Pachón DM, Robert V, Röhrig J, Ruller R, Salamov A, Salih NS, Samson RA, Sándor E, Sanguinetti M, Schütze T, Sepčić K, Shelest E, Sherlock G, Sophianopoulou V, Squina FM, Sun H, Susca A, Todd RB, Tsang A, Unkles SE, van de Wiele N, van Rossen-Uffink D, de Castro Oliveira JV, Vesth TC, Visser J, Yu J-H, Zhou M, Andersen MR, Archer DB, Baker SE, Benoit I, Brakhage AA, Braus GH, Fischer R, Frisvad JC, Goldman GH, Houbraken J, Oakley B, Pócsi I, Scazzocchio C, Seiboth B, vanKuyk PA, Wortman J, Dyer PS, Grigoriev IV (2017) Comparative genomics reveals high biological diversity and specific adaptations in the industrially and medically important fungal genus Aspergillus. Genome Biology 18. https://doi.org/10.1186/s13059-017-1151-0

Gnerre S, MacCallum I, Przybylski D, Ribeiro FJ, Burton JN, Walker BJ, Sharpe T, Hall G, Shea TP, Sykes S, Berlin AM, Aird D, Costello M, Daza R, Williams L, Nicol R, Gnirke A, Nusbaum C, Lander ES, Jaffe DB (2011) High-quality draft assemblies of mammalian genomes from massively parallel sequence data. Proceedings of the National Academy of Sciences 108: 1513–1518. https://doi.org/10.1073/pnas.1017351108

Grigoriev IV, Nikitin R, Haridas S, Kuo A, Ohm R, Otillar R, Riley R, Salamov A, Zhao X, Korzeniewski F, Smirnova T, Nordberg H, Dubchak I, Shabalov I (2014) MycoCosm portal: gearing up for 1000 fungal genomes. Nucleic Acids Research 42: D699–D704. https://doi.org/10.1093/nar/gkt1183

Koch RA, Yoon GM, Aryal UK, Lail K, Amirebrahimi M, LaButti K, Lipzen A, Riley R, Barry K, Henrissat B, Grigoriev IV, Herr JR, Aime MC (2021) Symbiotic nitrogen fixation in the reproductive structures of a basidiomycete fungus. Current Biology 31: 3905-3914.e6. https://doi.org/10.1016/j.cub.2021.06.033

Martin J, Bruno VM, Fang Z, Meng X, Blow M, Zhang T, Sherlock G, Snyder M, Wang Z (2010) Rnnotator: an automated de novo transcriptome assembly pipeline from stranded RNA-Seq reads. BMC Genomics 11: 663. https://doi.org/10.1186/1471-2164-11-663

Mondo SJ, Dannebaum RO, Kuo RC, Louie KB, Bewick AJ, LaButti K, Haridas S, Kuo A, Salamov A, Ahrendt SR, Lau R, Bowen BP, Lipzen A, Sullivan W, Andreopoulos BB, Clum A, Lindquist E, Daum C, Northen TR, Kunde-Ramamoorthy G, Schmitz RJ, Gryganskyi A, Culley D, Magnuson J, James TY, O'Malley MA, Stajich JE, Spatafora JW, Visel A, Grigoriev IV (2017) Widespread adenine N6-methylation of active genes in fungi. Nature Genetics 49: 964–968. https://doi.org/10.1038/ng.3859

Mondo SJ, He G, Sharma A, Ciobanu D, Riley R, Andreopoulos WB, Lipzen A, Kuo A, LaButti K, Pangilinan J, Salamov A, Salamon H, Shu L, Gladden J, Magnuson J, Aime MC, O'Malley R, Grigoriev IV (2025) Consecutive low-frequency shifts in

A/T content denote nucleosome positions across microeukaryotes. iScience 28: 112472. https://doi.org/10.1016/j.isci.2025.112472

Nemri A, Saunders DGO, Anderson C, Upadhyaya NM, Win J, Lawrence G, Jones D, Kamoun S, Ellis J, Dodds P (2014) The genome sequence and effector complement of the flax rust pathogen Melampsora lini. Frontiers in Plant Science 5. https://doi.org/10.3389/fpls.2014.00098

Padamsee M, Kumar TKA, Riley R, et al (2012) The genome of the xerotolerant mold Wallemia sebi reveals adaptations to osmotic stress and suggests cryptic sexual reproduction. Fungal Genet Biol 49:217–226. https://doi.org/10.1016/j.fgb.2012.01.007

Paul D, Magbanua Z, Arick M, et al (2014) Genome Sequence of the Oleaginous Yeast Rhodotorula glutinis ATCC 204091. Genome Announc 2:e00046-14. https://doi.org/10.1128/genomeA.00046-14

Toome M, Ohm RA, Riley RW, et al (2014)a Genome sequencing provides insight into the reproductive biology, nutritional mode and ploidy of the fern pathogen Mixia osmundae. New Phytologist 202:554–564. https://doi.org/10.1111/nph.12653

Toome M, Kuo A, Henrissat B, et al (2014)b Draft Genome Sequence of a Rare Smut Relative, Tilletiaria anomala UBC 951. Genome Announcements 2:10.1128/genomea.00539-14. https://doi.org/10.1128/genomea.00539-14
